# Regulatory hotspot on the influenza A virus polymerase revealed through the structure of the NEP-polymerase complex

**DOI:** 10.1126/sciadv.aeb4073

**Published:** 2026-01-23

**Authors:** Alison Rep, Fangzheng Wang, Kuang-Yu Chen, Loïc Carrique, Jane Sharps, Jonathan M. Grimes, Ervin Fodor

**Affiliations:** ^1^ Sir William Dunn School of Pathology, University of Oxford, Oxford, UK.; ^2^ Division of Structural Biology, Nuffield Department of Medicine, Centre for Human Genetics, University of Oxford, Oxford, UK.

## Abstract

Influenza A virus (IAV) transcribes and replicates its segmented RNA genome in the host nucleus within viral ribonucleoproteins (vRNPs), which are exported for virion assembly. The nuclear export protein (NEP) is essential for this process and also regulates viral RNA synthesis, implicating a direct interaction with the viral RNA polymerase. Here, we present a 2.5-Å cryogenic electron microscopy structure of NEP bound to the IAV polymerase and demonstrate that NEP alone is sufficient to promote vRNP export, with the viral matrix protein 1 enhancing export efficiency. NEP forms a four-helix bundle that binds at the interface of the PA C-terminal domain and PB1 N terminus of the polymerase. The NEP binding site at this interface overlaps with those for the host ANP32 and the C-terminal domain of RNA polymerase II, indicating that it functions as a regulatory hotspot coordinating transitions of the viral polymerase between RNA synthesis and nuclear export, revealing a critical layer of control in the IAV replication cycle.

## Teaser

Structural studies reveal how influenza virus switches between making new RNA genomes and exporting them from the host nucleus.

## INTRODUCTION

Influenza A viruses (IAVs) package their negative sense, segmented RNA genome into viral ribonucleoproteins (vRNPs). Each vRNP comprises a genomic viral RNA (vRNA) segment bound at its 5′ and 3′ ends by a single copy of the viral RNA-dependent RNA polymerase. This polymerase is a heterotrimer composed of the polymerase basic 1 (PB1), polymerase basic 2 (PB2), and polymerase acidic (PA) subunits (*1*). The viral nucleoprotein (NP), an essential structural protein involved in viral replication, oligomerizes along the remaining length of the vRNA (*2*). Viral transcription and replication occur within the nucleus of the infected cell in the context of vRNPs (*3*, *4*).

Interaction of the polymerase with the C-terminal domain (CTD) of host RNA polymerase II (Pol II) enables cap-snatching, a process in which the viral polymerase cleaves the 5′ end of a nascent host RNA transcript and uses the resulting capped RNA fragment to prime viral mRNA transcription (*5*, *6*). The viral transcripts are exported from the nucleus to the cytoplasm where they are translated into viral proteins (*7*). Several of these viral proteins, including polymerase subunits and NP, contain nuclear localization signals and are imported into the nucleus to participate in viral replication (*8*).

Replication of vRNA occurs via a complementary RNA (cRNA) intermediate (*3*, *9*), which is bound by NP and the viral polymerase, forming a cRNP (*3*, *10*, *11*). IAV genome replication relies on polymerase dimerization (*12*–*15*). An asymmetric polymerase dimer is involved in both steps of genome replication, with one polymerase acting as the replicating polymerase, while the second polymerase encapsidates the nascent RNA product. The host chaperone acidic nuclear phosphoprotein 32 (ANP32), which is essential for viral replication, promotes this asymmetric polymerase dimerization, enabling replication to proceed in a primer-independent manner (*9*).

The IAV genome comprises eight RNA segments that encode 10 major viral proteins. Segment 8, the nonstructural (NS) segment, produces the NS1 mRNA product and a splice variant encoding the nuclear export protein (NEP) (also known as NS2) (*16*). NS1 has been demonstrated to have an immunomodulatory role during infection, and NEP has been implicated in the export of vRNPs from the nucleus via exportin-1 (XPO1) (*16*, *17*). In addition to its role in nuclear export, NEP has been demonstrated to affect the levels of viral RNA species throughout infection (*18*–*21*). In vRNP reconstitution assays, expression of NEP led to changes in the abundance of the three viral RNA products to levels similar to those observed during viral infection (*20*). At low levels, NEP is thought to positively regulate viral genome replication while negatively affecting viral transcription, whereas at high expression levels, NEP inhibits the production of all viral RNA products (*18*–*20*, *22*).

Sequence analysis of NEP predicts two N-terminal alpha helices (N1 and N2), each containing a nuclear export signal (NES1 and NES2), and two C-terminal α helices (C1 and C2) (*23*, *24*). NES1 has been functionally validated by substituting it into the HIV-1 Rev protein, replacing the Rev NES, where it restored Rev nuclear export ability (*24*). Furthermore, in a yeast two-hybrid system, NEP has been shown to interact with multiple cellular nucleoporins, including those that Rev was capable of binding (*24*). The mechanism of vRNP nuclear export remains debated, with various viral and host proteins implicated. NEP is proposed to interact with host XPO1 via the NES sequences and mediate XPO1 interactions with the vRNP (*23*–*26*). Early models proposed a daisy-chain interaction in which NEP binds vRNPs through matrix protein 1 (M1), that is bound to NP in vRNPs (*27*–*30*). However, a revised model proposes that NEP binds directly to the viral polymerase in the vRNP, in an M1-dependent manner, to bridge vRNP-XPO1 interactions (*21*). The C terminus of NEP has been proposed to bind to the PB2 subunit of the polymerase within the nuclear export complex (*21*). X-ray crystallography revealed that the C-terminal half of NEP comprises two antiparallel α helices and more recent cryogenic electron microscopy (cryo-EM) studies demonstrate that NEP consists of four helices that can form domain-swapped dimers in the context of a polymerase hexamer (*31*, *32*).

Despite extensive research on NEP function, several critical questions remain unanswered regarding the precise binding to the viral polymerase complex and the structural basis for its dual role in nuclear export and transcription and replication regulation. Here, we used high-resolution cryo-EM to visualize and characterize the direct interaction between NEP and the influenza viral polymerase. Our 2.5-Å-resolution structure reveals that the NEP forms specific contacts with both the CTD of PA (PA-C) and the N terminus of PB1 (PB1-N). Through structure-guided mutagenesis and functional assays, we identify critical NEP residues that mediate these interactions and demonstrate the formation of this interface in cells and its importance in regulating polymerase activity. We also demonstrate that NEP is the sole viral factor that the vRNP requires for nuclear export, but that M1 makes the process more efficient. These findings provide a molecular framework for understanding how NEP coordinates multiple essential functions during the influenza virus life cycle and offer potential targets for antiviral intervention.

## RESULTS

### NEP alone is sufficient for vRNP nuclear export

**Fig. 1.**
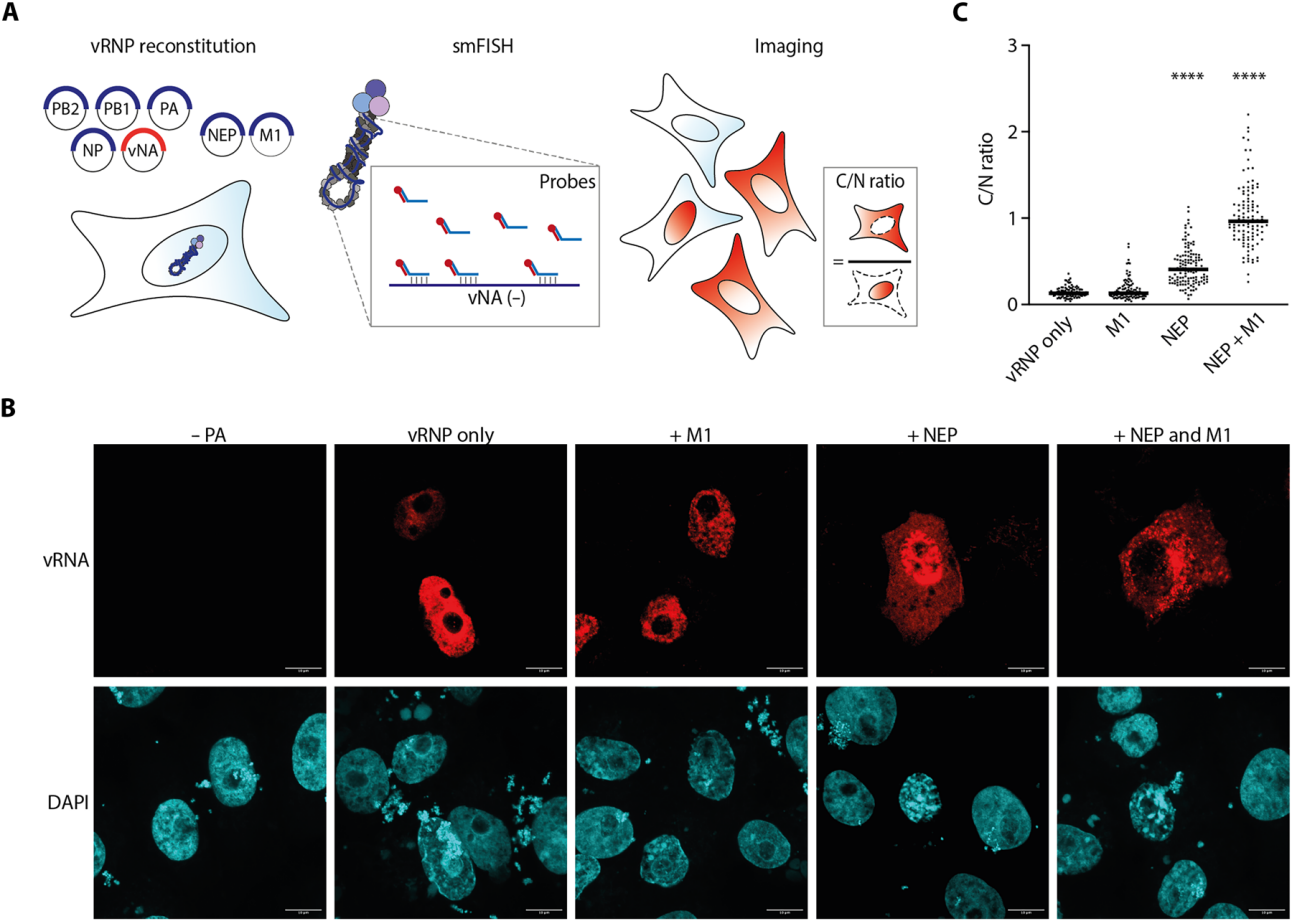
NEP is required for vRNP nuclear export. (**A**) Schematic of the vRNP reconstitution assay. Cells were transfected with plasmids expressing PB2, PB1, PA, NP, and NA vRNA, in the presence or absence of GFP-NEP and/or M1. PA was omitted as negative control (−PA). vRNP localization was assessed using smFISH probes targeting NA vRNA. Cellular and nuclear boundaries were delineated using GFP fluorescence and DAPI staining, respectively. The cytoplasmic-to-nuclear (C/N) ratio was calculated by dividing the mean fluorescence intensity in the cytoplasm by that in the nucleus. (**B**) Representative images of Vero E6 cells transfected with vRNP reconstitution components and fixed 24 hours post-transfection. NA vRNA signals are shown in the upper panels; DAPI staining of nuclei is shown in the lower panels. Scale bars, 10 μm. (**C**) Quantification of the C/N ratio from individual cells across two independent biological replicates: vRNP only (*n* = 100), M1 (*n* = 105), NEP (*n* = 132), and NEP + M1 (*n* = 109). The black lines represent medians. Statistical analysis was performed using nonparametric one-way analysis of variance (ANOVA) with multiple comparisons relative to the vRNP-only condition, ****P ≤ 0.0001.

To determine the minimal components required for vRNP nuclear export, single-molecule fluorescence in situ hybridization (smFISH) targeting vRNA was performed to assess the localization of vRNPs in the vRNP reconstitution system (Fig. 1A). To reconstitute vRNPs, we transfected cells with plasmids expressing the three polymerase subunits (PB1, PB2, and PA), NP, and the neuraminidase vRNA segment. A robust vRNA signal was observed in the nucleus, which was absent when the PA subunit was omitted, indicating that we are detecting vRNA replicated by the viral polymerase. In the absence of NEP, vRNA signals remained nuclear, indicating that vRNPs alone are unable to undergo nuclear export (Fig. 1B). Addition of M1 without NEP did not alter the localization of vRNPs. However, NEP alone was sufficient to trigger vRNP nuclear export of reconstituted vRNPs, as evidenced by the vRNA signal observed in the cytoplasm. Coexpression of NEP and M1 further increased the proportion of cytoplasmic vRNPs and led to the formation of distinct cytoplasmic puncta (Fig. 1, B and C). In the context of viral infection, the onset of vRNP nuclear export coincided with the accumulation of NEP (fig. S1). These results suggest that NEP is the minimal essential viral factor required for vRNP nuclear export, while M1 enhances this process and modulates the cytoplasmic localization of vRNPs following export.

### NEP binds the IAV polymerase at the interface of the PA-C and the PB1-N

**Fig. 2.**
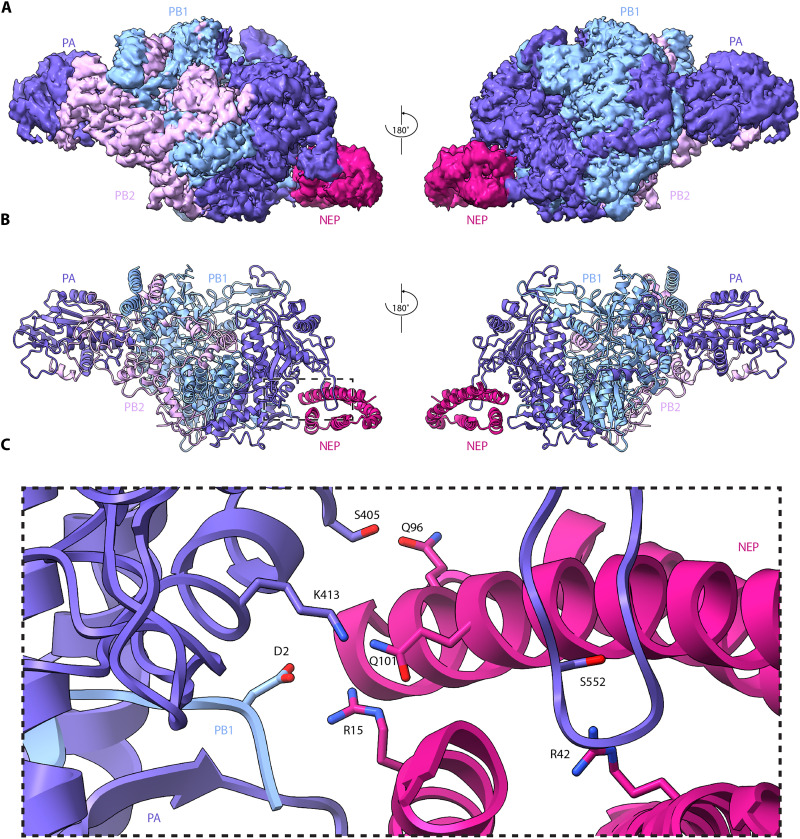
Structure of the pandemic A/Brevig Mission/1/18 (H1N1) IAV NEP-polymerase complex. (**A**) Cryo-EM map of the NEP-polymerase complex. (**B**) Cartoon model of the NEP-polymerase complex. Dashed rectangle denotes the close-up view shown in (C). (**C**) Close-up view of the NEP-polymerase interface demonstrating four pairs of interacting amino acid residues.

To investigate how NEP interacts with the IAV polymerase, we expressed the polymerase subunits (fig. S2, A and B) from the pandemic A/Brevig Mission/1/1918 (H1N1) strain (hereafter referred to as the 1918 virus) in insect cells, and NEP in bacteria. To assemble the NEP-polymerase complex for cryo-EM analysis, purified polymerase and NEP were mixed in a 1:5 molar ratio. Mass photometry analysis confirmed the importance of low salt for complex formation (fig. S2C). Analysis by cryo-EM revealed that the majority of 2D classes contained symmetric dimers of the IAV polymerase, consistent with previous observations for other IAV polymerases (*13*) (fig. S3). Following ab initio and heterogenous refinement, density corresponding to a symmetric dimer with two copies of NEP was observed. Each NEP molecule contacts only one polymerase monomer, indicating that binding of NEP is compatible with the polymerase dimer and that it does not induce its formation. Symmetry expansion was applied to increase particle numbers, before focusing the classification on a single copy of the polymerase and NEP. We obtained a final structure of a polymerase monomer bound to a single NEP molecule at a 2.53 Å resolution (Fig. 2A). The PB1 and PA polymerase subunits are well resolved, as are the PB2 N-terminal region (residues 1 to 248) and the PB2 nuclear localization signal domain (residues 692 to 753). However, no density is observed for the PB2 cap-binding, mid-link, and 627 domains. NEP is fully resolved except for the six N-terminal residues.

NEP binds at the interface of the PA-C and the PB1-N as a four-helix bundle (Fig. 2B). The interaction is mediated by four key residue pairs (Fig. 2C). R15 in the N1 helix of NEP forms a salt bridge with residue D2 of the PB1-N. In the N2 helix of NEP, R42 contacts PA residue S552, and in the NEP C2 helix, Q96 and Q101 interact with PA residues S405 and K413, respectively. The previously described phosphorylated triple serine motif at residues 23 to 25 of NEP (*33*) is exposed and points toward the PA-C, below the PB1-N, but does not contribute to the binding interface. The NEP C1 helix does not make any contacts with the polymerase and is oriented outward from the binding pocket formed by the PA-C and PB1-N, toward the PB1 finger and palm subdomains. The C1 and C2 helices are packed tightly, with most hydrophobic residues buried within the helical interface, consistent with the previously reported crystal structure of the NEP C-terminal region (*32*). Within the N terminus, the amphipathic nature is markedly reduced, and several hydrophobic residues of the N1 helix face the exterior, while others remain buried within the helical interface with N2. The NEP density is weakest at the turn between the two N-terminal helices (N1 and N2) and the two C-terminal helices (C1 and C2) (fig. S4), which corresponds to the region most distal from the NEP-polymerase interface. This likely reflects the flexibility of NEP at this region, enabling it to perform conformational changes required for interactions with XPO1.

We also solved the structure of NEP from the A/WSN/33 (H1N1) influenza virus bound to the 1918 polymerase (fig. S5) using the same methods as described above (fig. S6). The binding interface is identical to that in the 1918 NEP-polymerase structure, and NEP exhibits similar flexibility in the region most distal from the interface.

To address whether the observed NEP-polymerase interface is conserved across different influenza subtypes, we aligned the relevant sections of the sequence of PA-C/PB1-N and NEP of a selection of human, avian, equine, and swine IAVs belonging to different subtypes (fig. S7). We found that the interacting amino acids are highly conserved suggesting that the observed NEP-polymerase interaction is a conserved feature of all IAV subtypes.

### Cell-based validation of NEP-polymerase interface

**Fig. 3.**
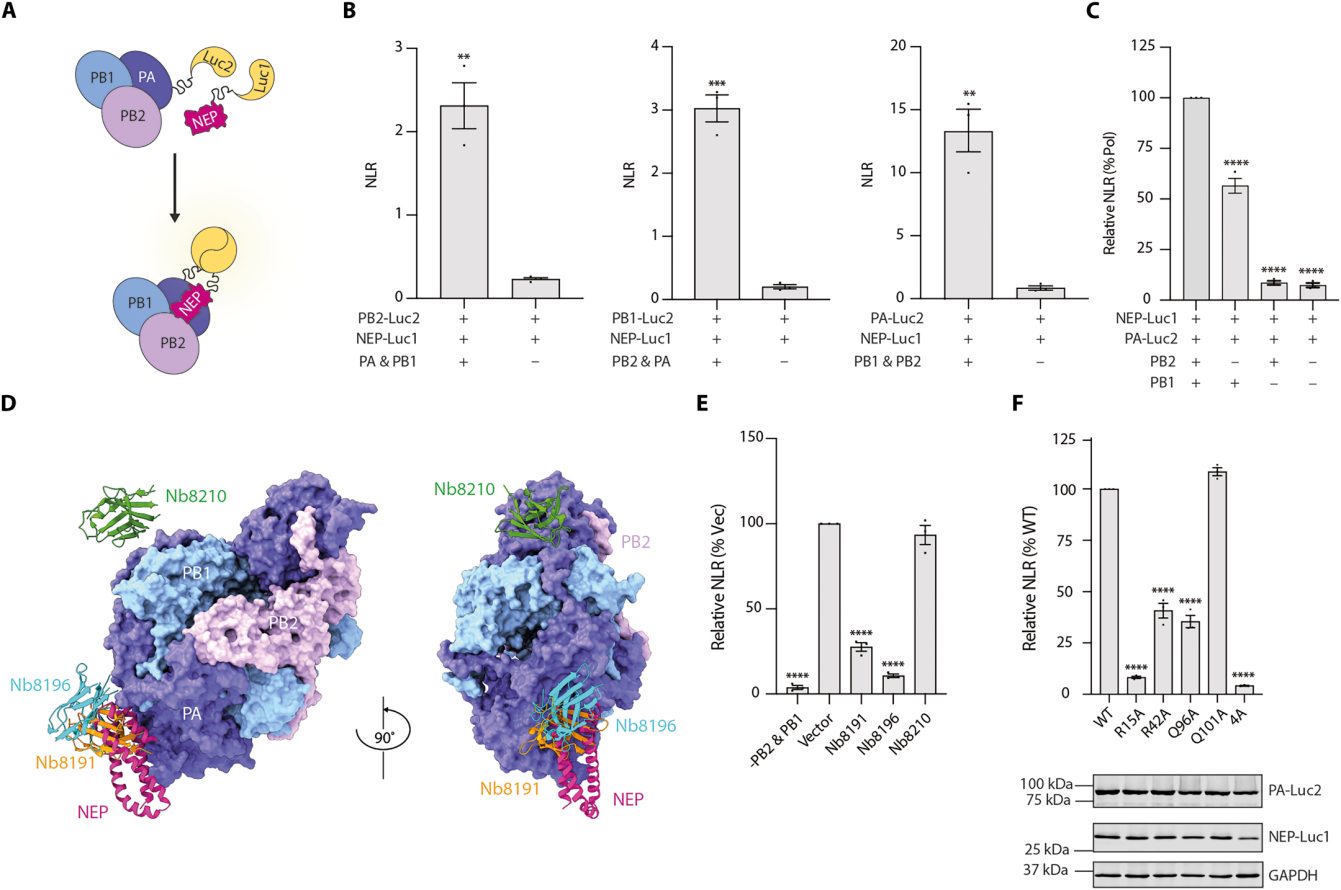
NEP interacts with the viral polymerase in cells via the NEP-polymerase interface observed by cryo-EM. (**A**) Split-luciferase complementation assay schematic. (**B**) HEK-293T cells were cotransfected with plasmids expressing NEP-Luc1 and the indicated luciferase-tagged polymerase subunit in the presence or absence of the remaining two polymerase subunits. Luminescence was measured 24 hours post-transfection and quantified as the normalized luminescence ratio (NLR). (**C**) NEP-polymerase interactions measured by split-luciferase assay using NEP-Luc1 and PA-Luc2 in the presence or absence of the full polymerase complex. Relative NLR was calculated by normalizing the NLR to the value in condition containing the full polymerase complex, set as 100%. (**D**) NEP-polymerase complex cryo-EM density map with superimposition of polymerase structures bound by Nb8191 (PDB 7NIR), Nb8196 (PDB 7NJ3), and Nb8210 (PDB 7NKR). (**E**) Effect of nanobodies on NEP-polymerase interactions measured by split-luciferase assay. Indicated nanobodies were coexpressed in the split-luciferase system. NLR values were normalized to vector control, set as 100%. (**F**) Effect of NEP interface mutations on NEP-polymerase interactions determined by split-luciferase assay. The 4A mutant was analyzed in a separate experiment with wild-type (WT) NEP. NLR values were normalized to the value of WT NEP, set as 100% within each experiment. The 4A mutant was then plotted alongside the individual interface mutants for the clarity of visualization. Western blots show expression levels of WT and interface mutants NEP-Luc1 and PA-Luc2, with GAPDH as a loading control. Graphs represent the NLR or relative NLR of three biological replicates (mean ± SEM, *n* = 3). Significance was determined using either two-tailed, unpaired Student’s *t* test (B) or ordinary one-way ANOVA with multiple comparisons relative to (C) full polymerase complex, (E) Vector, (F) WT, **P ≤ 0.01, ***P ≤ 0.001, and ****P ≤ 0.0001.

To validate our structural observation that NEP and viral polymerase form a complex, we used a split-luciferase complementation assay to monitor NEP-polymerase interactions in cells, measuring the interaction-mediated normalized luminescence ratio (NLR) (*34*). The N- and C-terminal halves of the *Gaussia princeps* luciferase were fused to the C terminus of NEP and each individual polymerase subunit, respectively (Fig. 3A). Coexpression of luciferase-tagged NEP and one luciferase-tagged polymerase subunit, together with the remaining two untagged polymerase subunits, yielded detectable luminescence signals. Among the three combinations tested, the luciferase-tagged NEP and PA generated the highest NLR (Fig. 3B). Therefore, we selected the combination of tagged NEP and PA subunit for further analysis.

NEP-polymerase interaction strength was markedly reduced when any individual polymerase subunit was omitted, confirming that the full polymerase complex is required for robust NEP binding (Fig. 3C). Notably, exclusion of PB2 subunit had a milder effect compared with the omission of PB1 subunit, consistent with our structural observation that the PB2 subunit does not directly contribute to the NEP-polymerase interface. To further confirm that our split-luciferase assay is measuring the same interface observed by cryo-EM, we tested the effect of previously reported nanobodies that bind to different regions of the PA subunit (*35*) on the NEP-polymerase interaction. Nanobodies Nb8191 and Nb8196, which bind to overlapping sites with NEP on PA (Fig. 3D), markedly diminished NEP-polymerase interactions (Fig. 3E). In contrast, Nb8210, which binds specifically to the PA linker region not directly involved in the NEP-polymerase interface, had no effect on NEP-polymerase interactions. These results suggest that the split-luciferase assay captures the same NEP-polymerase interface as we observed in the structure.

We next used this validated split-luciferase assay to assess the contributions of NEP interface residues (R15, R42, Q96, and Q101) to polymerase binding. Alanine substitutions of R15, R42, and Q96 substantially reduced NEP-polymerase interactions, whereas mutation of Q101 did not affect the interactions (Fig. 3F). Of note, the R15A and the quadruple mutant (R15A/R42A/Q96A/Q101A, referred to as 4A) almost completely abolished binding, although the expression level of the 4A mutant was somewhat reduced compared to wild-type or other NEP interface mutants. Together, these results demonstrate that the NEP-polymerase complex observed by cryo-EM also forms in cells and that this interaction is primarily mediated by the PB1 subunit and N-terminal helix of NEP.

### NEP-polymerase interface mutants retain vRNP nuclear export ability

**Fig. 4.**
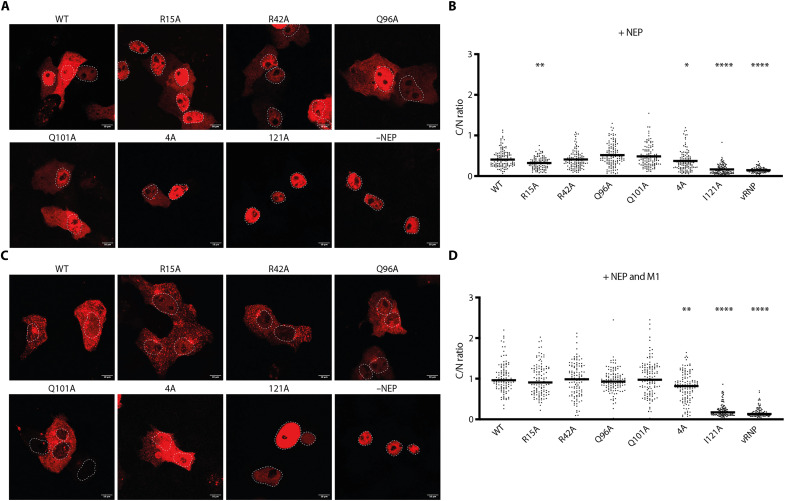
Effect of NEP mutants on vRNP localization. (**A**) Vero E6 cells were transfected with vRNP reconstitution components in the presence of GFP-tagged wild-type (WT) NEP or interface mutant NEPs or the absence of NEP (−NEP) and fixed 24 hours post-transfection. vRNP localization was assessed using smFISH probes targeting NA vRNA. Nuclear boundaries were delineated by DAPI staining (dashed lines; see fig. S8 for corresponding DAPI staining and GFP signal). Scale bars, 10 μm. (**B**) Quantification of cytoplasmic-to-nuclear (C/N) vRNA signal intensity ratios corresponding to (A). The black lines represent medians. (**C**) Same as in (A), with the addition of M1 expression. (**D**) Quantification of cytoplasmic-to-nuclear (C/N) vRNA signal intensity ratios corresponding to (C). Cell counts per condition for (B) [NEP only]: WT (*n* = 132), R15A (*n* = 114), R42A (*n* = 119), Q96A (*n* = 122), Q101A (*n* = 116), 4A (*n* = 117), 121A (*n* = 112), and vRNP only (*n* = 100). For (D) [NEP + M1]: WT (*n* = 109), R15A (*n* = 117), R42A (*n* = 114), Q96A (*n* = 123), Q101A (*n* = 123), 4A (*n* = 133), 121A (*n* = 126), and vRNP only (*n* = 105). Quantitation was performed from two independent biological replicates. Statistical analysis was performed using nonparametric one-way ANOVA with multiple comparisons to WT NEP condition, *P ≤ 0.05, **P ≤ 0.01, and ****P ≤ 0.0001.

Having resolved the structure of the NEP-polymerase complex, we next investigated whether the observed interface is directly involved in vRNP nuclear export. We conducted smFISH as described above, replacing wild-type NEP with single amino acid interface mutants or the quadruple mutant. We found that expression of NEP interface mutants did not drastically alter the localization of vRNPs compared to wild-type NEP, and nuclear export still occurred for all mutants, albeit to varying degrees (Fig. 4, A and B). A previous mutational screen demonstrated that maintaining hydrophobicity at residue 121 of NEP is critical for virus viability (*18*). To determine whether this residue also contributes to vRNP nuclear export, we tested the NEP I121A mutant. smFISH revealed that NEP I121A led to a strictly nuclear vRNP phenotype, indicating a failure to support vRNP nuclear export. To ensure this defect was not due to altered NEP localization, we assessed all NEP mutants using green fluorescent protein (GFP)–tagged constructs. For all mutants, including NEP I121A, signal was observed both in the cytoplasm and nucleus, indicating that mutations did not alter NEP localization (fig. S8). Coexpression of M1 with the NEP mutants increased the proportion of vRNPs in the cytoplasm for all NEP mutants except NEP I121A, which retained exclusive nuclear vRNP localization (Fig. 4, C and D). Overall, these data show that the polymerase-binding interface of NEP is not strictly required for vRNP nuclear export and support the notion that additional binding partners or structural rearrangements may be required to stabilize the NEP-polymerase complex in the context of vRNP nuclear export. The severe export defect observed with NEP I121A underscores the importance of the NEP C terminus in this process and suggests that the interface captured in our structure likely represents a transitional state toward the fully assembled nuclear export complex.

### Binding of NEP to polymerase inhibits viral RNA synthesis in a dose-dependent manner

**Fig. 5.**
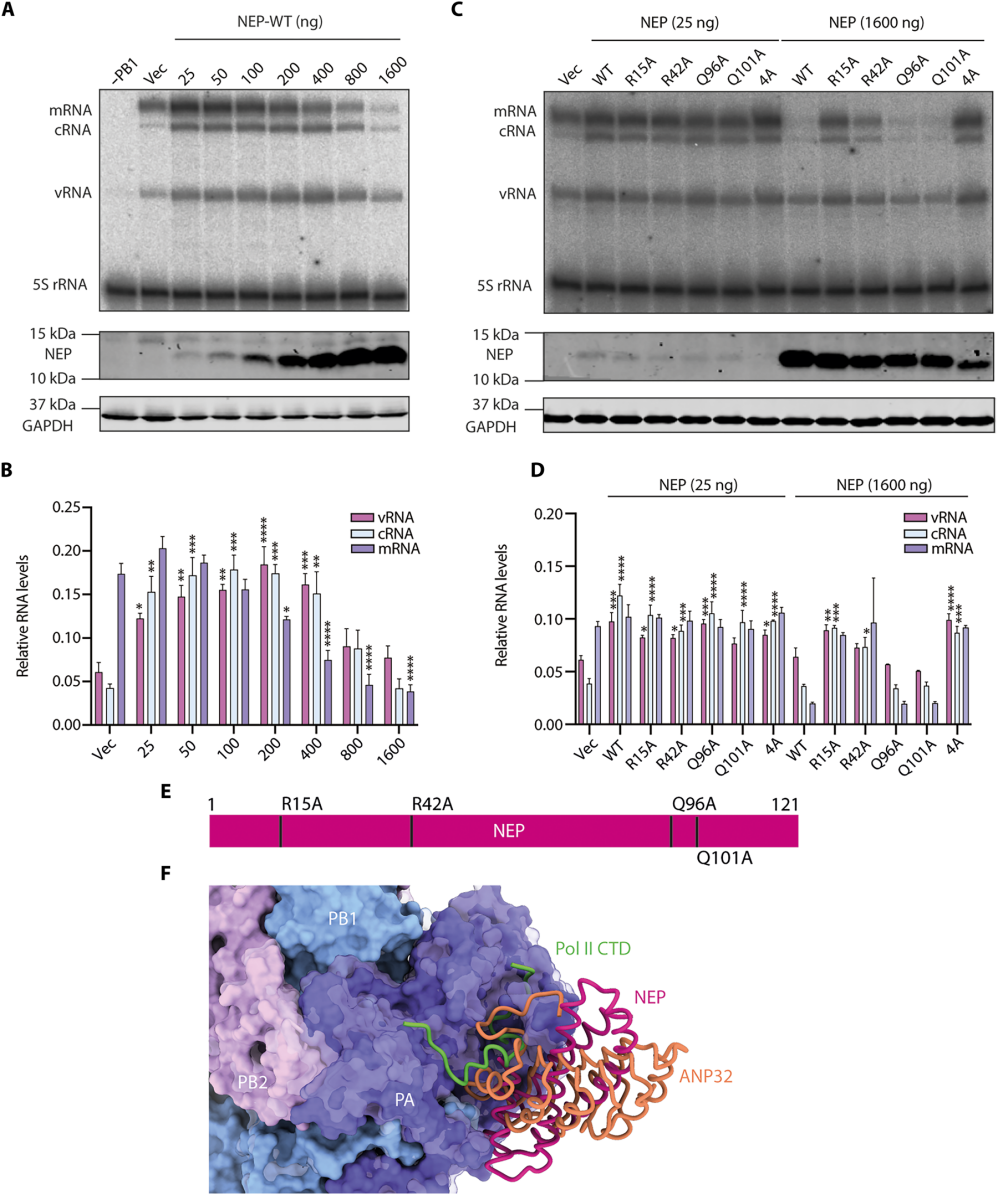
Effect of NEP mutants on viral RNA synthesis. (**A**) Primer extension analysis of viral RNAs from vRNP reconstitution assays in the presence of increasing concentrations of wild-type (WT) NEP or vector only (Vec). Omission of PB1 (−PB1) served as a negative control and 5*S* rRNA was used as a loading control. Western blotting using antibodies against NEP and GAPDH was conducted to assess protein levels of NEP and GAPDH (loading control). (**B**) Quantification of the viral RNA species from (A). Error bars represent the SEM (mean ± SEM, *n* = 4). Statistical significance was determined using ordinary one-way ANOVA with multiple comparisons to Vec condition, *P ≤ 0.05, **P ≤ 0.01, ***P ≤ 0.001, and ****P ≤ 0.0001. (**C**) Primer extension analysis of viral RNAs from vRNP reconstitution assays in the presence of low (25 ng) or high concentrations (1600 ng) of wild-type or indicated interface mutant NEPs or vector only (Vec). 5*S* rRNA was used as a loading control. Western blotting using antibodies against NEP and GAPDH was conducted to assess protein levels of NEP and GAPDH (loading control). (**D**) Quantification of the viral RNA species from (C). Error bars represent the standard error of the mean from three independent biological replicates (mean ± SEM, *n* = 3). Statistical significance was determined using ordinary one-way ANOVA with multiple comparisons to Vec condition, *P ≤ 0.05, **P ≤ 0.01, ***P ≤ 0.001, and ****P ≤ 0.0001. (**E**) Schematic representation of NEP protein depicting interface point mutations. (**F**) Cryo-EM model of the NEP-polymerase complex with the polymerase in surface representation and the NEP modeled in cartoon representation with superimposition of cartoon models ANP32B (PDB 8R1L) and Pol II CTD peptide mimic (PDB 8R60).

Knowing that NEP regulates influenza virus viral transcription and replication (*19*, *20*), we tested whether the NEP-polymerase structure we observed is involved in NEP-mediated regulation of viral polymerase activity. We first assessed the effect of wild-type NEP on viral RNA synthesis using the vRNP reconstitution system, supplemented with increasing amounts of NEP. We found that NEP modulates replication (vRNA and cRNA) and transcription (mRNA) in a dose-dependent manner (Fig. 5, A and B). Low amounts of NEP strongly increased vRNA and cRNA levels with relatively minor effects on mRNA, whereas higher NEP levels progressively reduced RNA levels. We then tested the effect of NEP interface mutants on viral RNA synthesis. At low doses, all interface mutants enhanced RNA synthesis comparably to wild-type NEP (Fig. 5, C and D). However, at high doses, their inhibitory effects varied broadly in accordance with their ability to interact with the polymerase. Specifically, the R15A and 4A mutants, which were unable to bind the polymerase, no longer inhibited RNA synthesis, whereas the remaining mutants showed an intermediate effect (R42A) or retained their inhibitory effect (Q96A and Q101A). Although the Q96A mutant showed a reduced ability to interact with the polymerase in the split-luciferase complementation assay, it was still able to efficiently inhibit RNA synthesis. This suggests that, in the context of a reconstituted vRNP, the interaction between the polymerase and NEP Q96A may be stabilized by additional interactions with NP and/or RNA, factors not present in the split-luciferase complementation assay. Consistent with these observations, the previously reported NEP I121A mutant (*31*) showed no reduction in polymerase interaction and retained its inhibitory effect at high levels, while losing its ability to enhance vRNA and cRNA synthesis at low levels (fig. S9). When combined with the R15A mutation, the double R15A/I121A mutant was no longer able to interact with the polymerase or inhibit polymerase activity at high levels, recapitulating the dominant negative phenotype of the interface mutant R15A. Overall, these data indicate that the interactions observed at the NEP-polymerase interface (Fig. 5E) are required for the inhibitory, but not the stimulatory, effect of NEP on RNA synthesis, and support the conclusion that the inhibitory activity of NEP at high doses is mediated by direct NEP-polymerase interactions.

To address the mechanism by which high levels of NEP result in viral RNA synthesis inhibition, we compared our NEP-polymerase complex structure with that of the IAV transcriptase and replicase revealing that the NEP binding site at the PA-C/PB1-N interface overlaps with the bindings sites for Pol II CTD and ANP32 proteins, factors essential for transcription and genome replication, respectively (Fig. 5F). This suggests that NEP inhibits viral RNA synthesis by competitively binding at the PA-C/PB1-N interface. Our data demonstrate that NEP-polymerase interactions lead to inhibition, rather than stimulation, of viral RNA synthesis and that this inhibition is dependent on the structural interface identified in our study and may occur through blocking interactions with Pol II CTD and ANP32.

## DISCUSSION

Our study provides insights into the multifunctional roles of IAV NEP in viral replication, particularly in viral RNA synthesis and vRNP nuclear export. Using a combination of structural, biochemical, and cellular approaches, we characterized the interaction between NEP and the viral polymerase complex and explored its functional implications. Specifically, we solved cryo-EM structures of NEP in complex with the viral polymerase, revealing that NEP adopts a four-helix bundle structure and binds to the polymerase at the interface of the PA-C domain and the PB1-N. We further demonstrated that this interaction occurs in cells and depends on the presence of the complete heterotrimeric polymerase complex. Nanobodies and interface mutations that disrupt the NEP-polymerase interface reduced this interaction in cells, confirming that the interface observed in the cryo-EM structure is functionally relevant in a cellular context. The functional importance of this binding site is further supported by findings from Keown *et al.* (*35*). In the context of viral infection, nanobody Nb8191, which occludes NEP binding to the polymerase, caused a pronounced reduction in viral titers across multiple time points, whereas Nb8210, which does not interfere with the NEP-polymerase interaction, had minimal or no effect. These observations independently validate the importance of the NEP-polymerase interface for efficient viral replication.

**Fig. 6.**
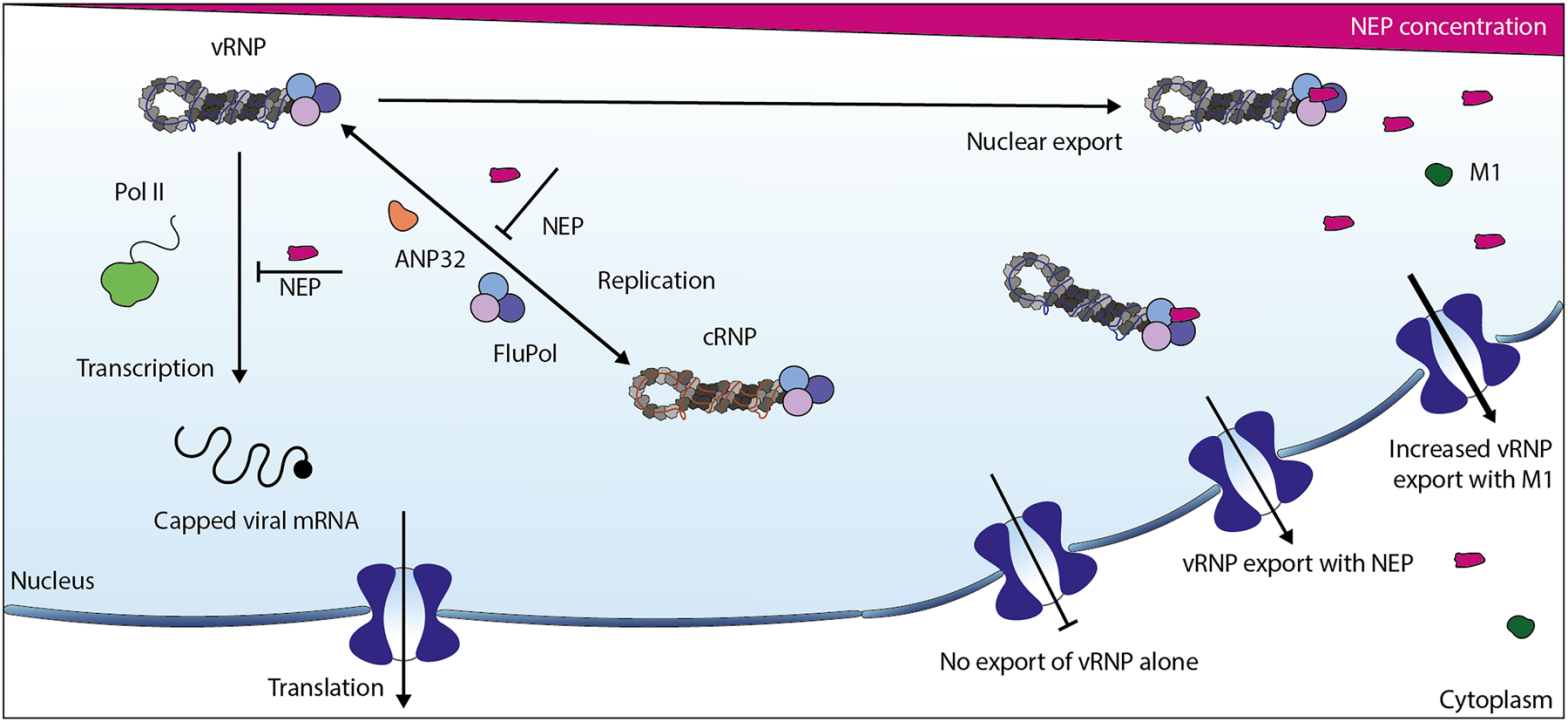
Roles of NEP in vRNP nuclear export and regulation of viral RNA synthesis. NEP, by competing with Pol II CTD and ANP32 for polymerase binding, inhibits viral transcription and genome replication, respectively, and promotes nuclear export. Without NEP, the vRNP cannot export, and with M1, export is more efficient.

To investigate the role of NEP-polymerase interactions, we performed vRNP reconstitution assays and found that while low levels of NEP promote viral RNA replication independently of the identified interface, high levels of NEP strongly inhibit viral RNA synthesis. Our structural analysis revealed that the NEP binding site at the PA-C/PB1-N interface on the polymerase overlaps with binding sites for other critical partners, including the CTD of host Pol II and ANP32 proteins (movie S1). This region of the polymerase may therefore serve as a regulatory hub that coordinates transitions between viral transcription, replication, and nuclear export in response to binding different viral and host factors (Fig. 6). The inhibition of viral RNA accumulation at high NEP levels in the vRNP reconstitution assay likely reflects NEP outcompeting other essential cofactors for binding at this hotspot. Supporting this interpretation, NEP interface mutants with reduced polymerase binding activity failed to inhibit viral RNA accumulation, while the stimulatory effect of low NEP expression persisted even when polymerase binding was disrupted, indicating that this proreplicative function occurs via a separate mechanism. While our data clearly demonstrate inhibition of viral RNA accumulation by high NEP levels in vRNP reconstitution assays the physiological relevance of this effect during infection remains to be determined.

Interactions of NEP, Pol II CTD, and ANP32 with the overlapping binding site at the PA-C/PB1-N interface may be shaped by local concentrations and compartmentalization within the nucleus rather than by direct competition. As NEP levels increase in the nucleus, NEP may occupy the PA-C/PB1-N site of the polymerase within newly assembled vRNPs as soon as they are released from the replication platform (*12*, *14*, *15*), before the vRNPs could encounter Pol II CTD or an ANP32-bound apo polymerase to engage in transcription or replication, respectively. The binding of NEP to the vRNP is likely further stabilized by other viral factors, such as M1, and by host XPO1/RanGTP, diverting vRNPs for nuclear export. In this model, NEP could mediate vRNP export without direct physical competition between partner proteins. At this stage, it remains difficult to predict how relative abundances and local distributions of ANP32, Pol II, and NEP influence the fate of a vRNP.

A recent study by Sun *et al.* (*31*) reported that NEP promotes the formation of a hexameric IAV RNA polymerase complex, potentially facilitating the transition from transcription to replication. In that hexameric structure, NEP forms a domain-swapped dimer and binds the polymerase at the same PA-C/PB1-N interface observed in our study. We searched our datasets for evidence of such a hexameric NEP-polymerase assembly but did not observe this complex. Sun *et al.* (*31*) also reported that interface-disrupting mutations, such as R15A, retained their replication-stimulatory function, consistent with our findings (*31*). This raises questions about how the hexameric structure promotes the transcription-to-replication switch. One possibility, also discussed by Sun *et al.* (*31*), is that an additional, as yet unidentified, NEP-polymerase interaction interface may mediate this regulatory effect. In agreement with this previous report, the I121A mutant was unable to support RNA replication at low levels (*19*). Alternatively, the increase in viral replication products observed at low NEP levels may reflect an indirect mechanism, potentially involving NEP-mediated modulation of the cellular environment to favor viral genome replication (*36*, *37*).

Our structure also has implications for understanding of adaptive mutations acquired during avian IAV transmission into humans. Avian IAV polymerases often show reduced activity in human cells, with adaptive mutations such as the classical PB2 E627K enhancing viral replication (*38*–*42*). In H5N1 strains lacking E627K, adaptive mutations such as M16I and Y41C have been reported in human isolates (*22*). These residues are neighbor to R15 and R42, which are critical contributors to the NEP-polymerase interface we observe in our structure. Another adaptive mutation to overcome avian polymerase restriction in humans is the T552S mutation within the PA subunit (*43*). In human strains of IAV, 552 is a highly conserved serine residue (*43*, *44*). In our structure, PA S552 makes direct contacts with NEP, further implicating NEP as a regulator of polymerase activity. Further work will be required to determine whether adaptive NEP mutations exert their effect through modulating NEP-polymerase interactions as observed in our structure, affect other functional interfaces or contribute to host adaptation through indirect mechanisms.

Our data also revealed that, in contrast to previous reports (*31*, *45*, *46*), NEP alone is sufficient to enable vRNP nuclear export. However, coexpression of M1 strongly enhanced vRNP cytoplasmic accumulation and shifted the cytoplasmic distribution of vRNPs from a diffuse to a more concentrated pattern. M1 has been proposed to form part of the vRNP nuclear export complex and is known to interact with NEP through residue W78 (*21*, *32*). In our structure, W78 is located on the outward-facing C1 helix and does not contact the polymerase. Attempts to incorporate M1 into our NEP-polymerase complex were unsuccessful, possibly due to the propensity of M1 to self-assemble into multiple oligomeric states (*47*–*49*). Currently, it remains unclear whether M1 participates directly in the export complex, as proposed previously, or it acts in the cytoplasm by binding and retaining exported vRNPs, enhancing their cytoplasmic accumulation. Together, these observations suggest that while M1 modulates the efficiency and spatial organization of vRNP export, NEP provides the minimal viral molecular machinery required to drive vRNP nuclear export.

NEP-polymerase interface mutations had no major impact on the ability of NEP to promote vRNP nuclear export. These data suggest that additional factors may contribute to the stabilization of the NEP-polymerase complex in the context of vRNP nuclear export. The above discussed M1 could contribute to the stabilization, and binding of XPO1/RanGTP could further support complex formation. Furthermore, structural analysis indicates that a conformational rearrangement of NEP is likely required for export function: Both NES1 and NES2 reside in the N-terminal helices, which in our structure, face the polymerase and are therefore inaccessible to XPO1. Since these helices also mediate the primary polymerase interaction, their release may depend on additional contacts involving the NEP C terminus, either anchoring NEP to the vRNP or bridging to the polymerase to expose the NESs. The strong nuclear retention of vRNPs observed with the NEP I121A mutant supports a critical role for the NEP C terminus in nuclear export. Further studies will be necessary to define these interactions and their dynamics within the full vRNP complex.

In summary, our study reveals a previously uncharacterized interaction between NEP and the influenza virus polymerase, which likely represents a transitional step in the assembly of the nuclear export complex. This interface may facilitate the termination of viral RNA synthesis and the initial recruitment of export factors, with full NES exposure and XPO1 engagement requiring further stabilization by host or viral components. The profound nuclear export defect caused by the NEP I121A mutation highlights an essential role for the NEP C terminus in linking the polymerase to the broader vRNP or export machinery. Our structural data identify a conserved binding hotspot at the PA-C/PB1-N interface that serves as a regulatory hub, coordinating access by multiple partner proteins, including Pol II CTD, ANP32, and NEP, at distinct stages of the viral replication cycle. Binding of different factors at an overlapping site of the viral polymerase suggests a finely tuned regulatory mechanism of transition from transcription to genome replication and vRNP nuclear export. Together, these findings not only advance our understanding of influenza virus RNA regulation and genome export but also identify a regulatory hotspot within the polymerase that may serve as a target for antiviral drug development.

## MATERIALS AND METHODS

### Cells and viruses

Sf9 insect cells, sourced from the Division of Structural Biology (STRUBI), University of Oxford, were maintained in Sf-900 II serum-free medium (Gibco) and cultured at 27°C with shaking at 110 rpm. HEK-293T, Vero E6, and A549 cells, sourced from the Cell Bank of the Sir William Dunn School of Pathology, University of Oxford, were cultured in Dulbecco’s modified Eagle’s medium (Sigma-Aldrich) supplemented with 10% fetal bovine serum (Sigma-Aldrich). All cells were maintained at 37°C with 5% CO_2_. Influenza virus A/WSN/33 (H1N1) was generated using reverse genetics (*50*).

### Plasmids

Plasmids pCAGGS-PB2, pCAGGS-PB1, pCAGGS-PA, and pCAGGS-NP, encoding sequences of the A/WSN/33 (H1N1) virus, were provided by A. García-Sastre. Plasmids pcDNA-PB2, pcDNA-PB1, pcDNA-PA, pcDNA-NP, pcDNA-NEP, and pPOLI-NA-RT, also encoding WSN viral sequences, were generated as previously described (*20*, *51*, *52*). Site-directed mutagenesis was used to introduce mutations into the NEP gene. Plasmids pCAGGS-NEP (WT and mutants) and pCAGGS-M1 were generated by amplifying the genes from pcDNA-NEP (WT and mutants) and pPOLI-M-RT, respectively, and cloning into the pCAGGS vector using Not I and Xho I restriction sites. Plasmids used in split-luciferase complementation assay for measuring polymerase-NEP interactions were constructed by fusing the N-terminal (Luc1) and C-terminal (Luc2) fragments of *Gaussia* luciferase to NEP and individual viral polymerase subunits, respectively, with a GGGSGGGS linker in between. The Luc1 and Luc2 fragments were also cloned into empty pcDNA vector to serve as controls. Plasmids pCAGGS-GFP-NEP and the corresponding interface mutants were generated by inserting the enhanced GFP open reading frame at the N terminus of NEP using EcoR I and Not I restriction sites. Nanobodies Nb8191, Nb8196, and Nb8210 were expressed from mammalian expression plasmids as previously described (*35*). To generate plasmid pGEX-6P-1-NEP, encoding the NEP sequence from the 1918 or WSN virus, NEP sequences codon optimized for *Escherichia coli* expression (GenScript) were cloned into pGEX vector using Gibson assembly.

### smFISH assay of vRNA localization

Vero E6 cells or A549 cells were seeded on 13-mm glass coverslips in 24-well plates at 0.6 × 10^5^ cells per well. The next day, Vero E6 cells were transfected with 100 ng of each pCAGGS-PB2, pCAGGS-PB1, and pCAGGS-PA; 200 ng of pCAGGS-NP; 25 ng of pPOLI-NA-RT; and 50 ng of pCAGGS-GFP-NEP (WT and mutants) and pCAGGS-M1 plasmids, using Lipofectamine 3000 (Invitrogen). At 24 hours post-transfection, cells were fixed with 4% paraformaldehyde for 20 min at room temperature, followed by permeabilization with 70% ethanol at 4°C overnight (*53*). A549 cells were infected with A/WSN/33 (H1N1) influenza virus at a multiplicity of infection of three and fixed at different time points postinfection, followed by the same fixation and permeabilization procedure as described for Vero E6 cells. Probes against the NA vRNA of A/WSN/33 (H1N1) were designed using the Stellaris probe designer (table S1) (*54*). In addition, a 28-nucleotide shared sequence (5′-CCTCCTAAGTTTCGAGCTGGACTCAGAA-3′) was added to all probes at the 5′ end to allow binding of complementary fluorescent-labeled primers (FLAP) (*55*). Probes were annealed with FLAP primers at an equimolar ratio. The smFISH hybridization protocol was modified from Haralampiev *et al.* (*53*). In brief, cells were washed with phosphate-buffered saline (PBS) to remove ethanol, incubated with 80% formamide at 37°C for 10 min, and then rehydrated with 2× SSC buffer (300 mM NaCl and 30 mM sodium citrate pH 7.0) at room temperature for 10 min. Cells were incubated at 37°C with a freshly prepared hybridization solution containing 200 nM probes, 2× SSC, 10% formamide, 10% dextran sulfate, and 2 mM ribonucleoside vanadyl complex. After 4 hours of incubation, cells were washed twice with warm 2× SSC buffer containing 10% formamide at 37°C for 10 min. Last, mounting medium containing 4′,6-diamidino-2-phenylindole (DAPI) (Ibidi) was applied to the cells for nuclear staining.

### Confocal microscopy and image analysis

Images were acquired using the FV3000 confocal laser scanning microscope (Olympus) with a 60× objective and High-Sensitivity Spectral Detectors (HSSD) and Spinning Disk (SD) cameras to detect the fluorescent signal. Excitation lasers were set at 405 nm for the DAPI signal, 488 nm for the GFP signal, and 561 nm for the FISH signal. Each image was captured at a resolution of 310 nm/pixel with image dimension of 1024 by 1024 pixels and a volume of 6 μm with 1.2 μm z-steps. Images were processed using ImageJ and Cellpose software for analysis to define nuclear and cellular boundaries by DAPI staining and GFP fluorescence, respectively. The FISH signal was quantified by measuring the average fluorescence intensity.

### Recombinant protein expression and purification

The three subunits of influenza A/Brevig Mission/1/1918 (H1N1) virus (hereafter referred to as the 1918 virus) polymerase were coexpressed in Sf9 cells from codon-optimized genes using the MultiBac system, and the polymerase heterotrimer was purified as previously described (*35*). In brief, affinity purification of the polymerase heterotrimer was conducted using IgG Sepharose resin (GE Healthcare) followed by size exclusion chromatography (SEC) using a Superdex 200 Increase 10/300 GL column (GE Healthcare) equilibrated in polymerase SEC buffer [25 mM HEPES-NaOH (pH 7.5) 500 mM NaCl, 5% (v/v) glycerol, and 1 mM dithiothreitol]. NEP of the 1918 virus and WSN virus were each expressed in the pGEX-6P-1 expression vector with an N-terminal glutathione *S*-transferase (GST) tag followed by a PreScission 3C protease cleavage site and a 6– or 7–amino acid linker, respectively, in BL21 *E. coli* cells. Bacterial cell pellets were resuspended in wash buffer [50 mM tris-HCl (pH 7.5), 300 mM NaCl, 50% glycerol, and 1 mM dithiothreitol] with the addition of lysozyme (1 mg/ml) from chicken egg white (Sigma-Aldrich), one protease inhibitor cocktail tablet (Roche) per 100 ml resuspended culture, ribonuclease A (100 μg/ml), and sonicated for 30-s on and 30-s off and repeated three times. Sonicated lysate was clarified through centrifugation at 35,000*g* for 1 hour before incubation with Glutathione Sepharose 4B (GE Life Sciences) pre-equilibrated beads for 1 hour at 4°C. The beads were washed three times with wash buffer at 4°C before elution with 25 mM reduced glutathione for 30 min or cleavage with human rhinovirus 3C protease, produced in house, for 1 hour. The beads were cleared from the protein containing supernatant with a 1500*g* centrifuge step for 5 min at 4°C. The sample was concentrated using a centrifugal concentrator (Millipore) with a molecular weight cutoff less than half the size of the target proteins, before being subjected to SEC. SEC was performed using a Superdex 200 Increase 10/300 GL column (GE Healthcare) equilibrated in NEP SEC buffer [25 mM tris-HCl (pH 7.5), 300 mM NaCl, 25% glycerol, and 1 mM dithiothreitol].

### Cryo-EM sample preparation

Before grid making, either WSN NEP or 1918 NEP was mixed with 1918 polymerase in a 5:1 molar ratio, to obtain a final concentration of polymerase around 0.6 mg/ml in buffer containing 150 mM NaCl. Grids were prepared using a Vitrobot Mark IV (Thermo Fisher Scientific) at 100% relative humidity. For grid preparation for the WSN NEP and 1918 polymerase sample, Quantifoil Holey Carbon R2/1 200 mesh copper grids were glow discharged before applying 3.5 μl of sample and blotting for 5 s with a blot force of −15, followed by vitrification in liquid ethane. For grid preparation for the 1918 NEP and 1918 polymerase sample, Quantifoil Holey Carbon R2/1 200 mesh gold grids were glow discharged, before applying 3.5 μl of sample at around 0.3 mg/ml and blotted for 5 s, blot force −15, before vitrification in liquid ethane.

### Cryo-EM image collection

Cryo-EM data were collected at the Oxford Particle Imaging Centre (OPIC) using a 300-kV G3i Titan Krios microscope (Thermo Fisher Scientific) fitted with a SelectrisX energy filter and Falcon IVi direct electron detector. Automated data collection was set up in EPU 3.4, and movies were recorded in EER format. Data were collected using AFIS with a total dose of approximately 50 e^−^/Å^2^, a calibrated pixel size of 0.932 Å/pixel, and a 10 eV slit. Specific data collection parameters for both the 1918 NEP and 1918 polymerase samples and the WSN NEP and 1918 polymerase samples are provided in the Supplementary Materials (table S2).

### Cryo-EM data processing

All datasets were processed using CryoSPARC V4.2-4.7 (*56*). The EER format movies were fractionated into 60 frames without applying an upsampling factor. Preprocessing was performed using patch motion correction and patch-CTF estimation with default settings. Corrected micrographs with poor statistics were manually curated.

For the 1918 NEP and 1918 polymerase dataset, a first round of blob picking followed by 2D classification generated initial templates that were used for template picking. After 2D classification, well-resolved classes were selected, and two ab initio models were generated and further refined using heterogeneous refinement. Particles belonging to the high-resolution class were used to train a Topaz model and pick a new set of particles. These particles were directly classified using heterogeneous refinement with the previous ab initio models input twice. One good 3D class representing a dimeric polymerase population with weak density for NEP on both monomers was selected. A total of 428,000 particles were refined using NU-refinement with per-particle CTF refinement, leading to a 2.53-Å-resolution map after reference-based motion correction. To take advantage of the dimeric population, we performed NU-refinement imposing C2 symmetry followed by C2 symmetry expansion and particle subtraction of the density corresponding to one polymerase-NEP molecule. Using the 856,000 expanded particles, we performed local NU-refinement on the other polymerase-NEP molecule, followed by 3D classification without alignment using 20 classes and 10,000 particles per initial reconstruction with a mask focusing on the NEP molecule. Only one class containing 105,000 particles displayed strong density for NEP. This class was selected and refined using local NU-refinement, leading to a 2.53-Å-resolution map.

For the WSN NEP and 1918 polymerase dataset, a first round of blob picking followed by 2D classification generated initial templates that were used for template picking. After 2D classification, well-resolved classes were selected, and three ab initio models were generated and further refined using heterogeneous refinement. Particles belonging to the high-resolution class were used to train a Topaz model and pick a new set of particles. These particles were directly classified using heterogeneous refinement with the previous ab initio models input twice. Two good 3D classes representing a dimeric polymerase population with weak density for NEP on both monomers were selected. Particles belonging to the previous good class were added to them, removing duplicated particles closer than 60 Å. A total of 293,000 particles were refined using NU-refinement with per-particle CTF refinement, leading to a 2.8-Å-resolution map. To take advantage of the dimeric population, we performed NU-refinement imposing C2 symmetry followed by C2 symmetry expansion and particle subtraction of the density corresponding to one polymerase-NEP molecule. Using the 586,000 expanded particles, we performed local NU-refinement on the other polymerase-NEP molecule, leading to a 2.58-Å-resolution map, followed by 3D classification without alignment using 20 classes and 10,000 particles per initial reconstruction with a mask focusing on the NEP molecule. Only one class containing 33,000 particles displayed strong density for NEP. This class was selected and refined using local NU-refinement, leading to a 2.93-Å-resolution map.

### Structure determination and model refinement

Initial modelling was performed using PDB 8R1J by first fitting the entire replicase conformation. Initial fitting was performed using UCSF ChimeraX V1.9. In WinCoot 0.9.8.7 (*57*), the restraints module was used to generate restraints at 4.3 Å and allow flexible refinement to fit the main chain into density. Multiple cycles of manual adjustment in WinCoot, followed by real-space refinement in PHENIX, were used to improve model geometry. The final model geometry and map-to-model comparison were validated using PHENIX MolProbity (*58*). Structural analysis and figures were prepared using UCSF ChimeraX.

### Split-luciferase complementation assay

HEK-293T cells in 24-well plates were transiently transfected with 200 ng of each pcDNA-NEP-Luc1 (WT or mutants), pcDNA-PB2, pcDNA-PB1, and pcDNA-PA-Luc2 (unless otherwise indicated in the figures) using Lipofectamine 2000 (Invitrogen). The two negative controls, pcDNA-NEP-Luc1 + pcDNA-Luc2 and pcDNA-PA-Luc2 + pcDNA-Luc1, were included for calculation of normalized luminescence signal (NLR) as previously described (*34*). Cells were harvested 24 hours post-transfection and subjected to a luciferase assay using the *Renilla* Luciferase Assay System (Promega), following the manufacturer’s instructions. Relative light units were read by GloMax 20/20 Luminometer (Promega) (dataset S1).

### vRNP reconstitution assay

HEK-293T cells in 35-mm dish were transiently transfected with 125 ng of each pcDNA-PB2, pcDNA-PB1, and pcDNA-PA; 500 ng of pcDNA-NP; and 100 ng of pPOLI-NA-RT together with indicated amount of pCAGGS-NEP expressing plasmids using Lipofectamine 2000 (Invitrogen). At 24 hours post-transfection, total RNA from half of the transfected cells was harvested for primer extension analysis, and the other half were lysed for immunoblotting.

### RNA isolation and primer extension analysis

Total RNA was extracted from HEK-293T cells in 35-mm dish using 500 μl of TRI reagent (Sigma-Aldrich) according to the manufacturer’s instructions. Primer extension analysis was performed as previously described (*59*). Two micrograms of total RNA was reverse transcribed using SuperScript III reverse transcriptase (Invitrogen) with ^32^P-labeled NA specific primers. A primer targeting cellular 5*S* rRNA was included to detect 5*S* rRNA which serves as internal control. cDNA from reverse transcription was resolved using a 6% denaturing polyacrylamide gel electrophoresis containing 7 M urea in tris-borate-EDTA buffer. Radioactive signals were detected by a Fujifilm phosphor screen and imaged on FLA-5000 scanner (Fuji). ImageJ was used to quantify signals and values were first normalized to the signal derived from the 5*S* rRNA control and subsequently normalized to the sum of the total RNA signals from the same species of viral RNA in each experiment. The values for the “-PB1” control were subtracted from the sample values. Data were analyzed using Prism 10.5 (GraphPad).

### Immunoblotting

Half of the transfected cells from vRNP reconstitution assay were lysed with lysis buffer [50 mM HEPES-NaOH (pH 8.0), 150 mM NaCl, 25% glycerol, 0.5% NP40, and 1 mM β-mercaptoethanol] supplemented with Protease Inhibitor Cocktail (cOmplete Mini, Roche) for 1 hour on ice. Cell lysates were clarified by centrifugation at 17,000*g* for 10 min at 4°C. Sample loading buffer [4×, 500 mM tris-HCl (pH 6.8), 4% SDS, 40% glycerol, 40 mM dithiothreitol, and 0.01% bromophenol blue] was added and cell lysates were heated at 95°C for 10 min. Protein samples were resolved by tris-glycine SDS–polyacrylamide gel electrophoresis and transferred to nitrocellulose membranes (Amersham). Membranes were blocked with 5% (w/v) skimmed milk in tris-buffered saline containing 0.1% (v/v) Tween-20 (TBST) at room temperature for 1 hour and then incubated with primary antibodies at 4°C overnight. Primary antibodies against NEP (GTX125952, GeneTex), *Gaussia* luciferase (PA1-181, Invitrogen), and glyceraldehyde-3-phosphate dehydrogenase (GAPDH; 14C10, Cell Signaling Technology) were used. After three washes with TBST, membranes were incubated with IRDye secondary antibodies (LICORBIO) at room temperature for 1 hour and washed three times before imaging with Odyssey DLx Imaging System (LICORBIO).

### Mass photometry

Mass photometry measurements were performed using a TwoMP mass photometer (Refeyn) (*60*, *61*). Borosilicate microscope 24 by 50 mm coverslips (Menzel-Gläser) were prepared by sonication first in a 50% water-isopropanol mixture before, followed by Milli-Q water. They were dried using clean N_2_ gas to ensure complete drying before a CultureWell reusable 3 mm diameter by 1 mm depth gasket (Grace Bio-Labs) cleaned in the same manner as the coverslip was mounted onto the coverslip. Mass calibration was performed in both low-salt (PBS) and high-salt [25 mM HEPES-NaOH (pH 7.5), 500 mM NaCl, 5% glycerol, and 1 mM dithiothreitol] buffers, with a medium field of view and monitored for 60 s using the AquireMP software (Refeyn). For each condition, 5 μl of relevant buffer was used to find focus. To make the complexes, 1918 polymerase and WSN GST-tagged NEP were diluted in relevant buffers to a final concentration of 25 nM and mixed in a 1:1 ratio. Fifteen microliters of the mixture was added to the coverslip and a 60-s movie was recorded using the AquireMP software (Refeyn). Movies were processed and the mass estimation done using the relevant calibration in the DiscoverMP software (Refeyn).

### Multiple sequence alignment

Multiple sequence alignment was conducted using amino acid sequences of NEP, PB1, and PA downloaded from the GISAID database and using Clustal Omega (*62*). The results were visualized using ESPript 3.0 (*63*).

## References

[1] A. J. Te Velthuis, E. Fodor, Influenza virus RNA polymerase: Insights into the mechanisms of viral RNA synthesis. Nat. Rev. Microbiol. 14, 479–493 (2016). 27396566 10.1038/nrmicro.2016.87 PMC4966622

[2] A. J. Eisfeld, G. Neumann, Y. Kawaoka, At the centre: Influenza A virus ribonucleoproteins. Nat. Rev. Microbiol. 13, 28–41 (2015). 25417656 10.1038/nrmicro3367 PMC5619696

[3] E. Fodor, A. J. Te Velthuis, Structure and function of the influenza virus transcription and replication machinery. Cold Spring Harb. Perspect. Med. 10, a038398 (2020). 31871230 10.1101/cshperspect.a038398 PMC7334866

[4] J. M. Wandzik, T. Kouba, S. Cusack, Structure and function of influenza polymerase. Cold Spring Harb. Perspect. Med. 11, a038372 (2021). 32341065 10.1101/cshperspect.a038372 PMC8415296

[5] T. Krischuns, M. Lukarska, N. Naffakh, S. Cusack, Influenza virus RNA-dependent RNA polymerase and the host transcriptional apparatus. Annu. Rev. Biochem. 90, 321–348 (2021). 33770447 10.1146/annurev-biochem-072820-100645

[6] A. P. Walker, E. Fodor, Interplay between influenza virus and the host RNA polymerase II transcriptional machinery. Trends Microbiol. 27, 398–407 (2019). 30642766 10.1016/j.tim.2018.12.013 PMC6467242

[7] A. York, E. Fodor, Biogenesis, assembly, and export of viral messenger ribonucleoproteins in the influenza A virus infected cell. RNA Biol. 10, 1274–1282 (2013). 23807439 10.4161/rna.25356 PMC3817148

[8] E. C. Hutchinson, E. Fodor, Transport of the influenza virus genome from nucleus to nucleus. Viruses 5, 2424–2446 (2013). 24104053 10.3390/v5102424 PMC3814596

[9] Z. Zhu, E. Fodor, J. R. Keown, A structural understanding of influenza virus genome replication. Trends Microbiol. 31, 308–319 (2023). 36336541 10.1016/j.tim.2022.09.015

[10] F. T. Vreede, T. E. Jung, G. G. Brownlee, Model suggesting that replication of influenza virus is regulated by stabilization of replicative intermediates. J. Virol. 78, 9568–9572 (2004). 15308750 10.1128/JVI.78.17.9568-9572.2004 PMC506943

[11] A. York, N. Hengrung, F. T. Vreede, J. T. Huiskonen, E. Fodor, Isolation and characterization of the positive-sense replicative intermediate of a negative-strand RNA virus. Proc. Natl. Acad. Sci. U.S.A. 110, E4238–E4245 (2013). 24145413 10.1073/pnas.1315068110 PMC3831450

[12] L. Carrique, H. Fan, A. P. Walker, J. R. Keown, J. Sharps, E. Staller, W. S. Barclay, E. Fodor, J. M. Grimes, Host ANP32A mediates the assembly of the influenza virus replicase. Nature 587, 638–643 (2020). 33208942 10.1038/s41586-020-2927-z PMC7116770

[13] H. Fan, A. P. Walker, L. Carrique, J. R. Keown, I. Serna Martin, D. Karia, J. Sharps, N. Hengrung, E. Pardon, J. Steyaert, J. M. Grimes, E. Fodor, Structures of influenza A virus RNA polymerase offer insight into viral genome replication. Nature 573, 287–290 (2019). 31485076 10.1038/s41586-019-1530-7 PMC6795553

[14] B. Arragain, T. Krischuns, M. Pelosse, P. Drncova, M. Blackledge, N. Naffakh, S. Cusack, Structures of influenza A and B replication complexes give insight into avian to human host adaptation and reveal a role of ANP32 as an electrostatic chaperone for the apo-polymerase. Nat. Commun. 15, 6910 (2024). 39160148 10.1038/s41467-024-51007-3 PMC11333492

[15] E. Staller, L. Carrique, O. C. Swann, H. Fan, J. R. Keown, C. M. Sheppard, W. S. Barclay, J. M. Grimes, E. Fodor, Structures of H5N1 influenza polymerase with ANP32B reveal mechanisms of genome replication and host adaptation. Nat. Commun. 15, 4123 (2024). 38750014 10.1038/s41467-024-48470-3 PMC11096171

[16] D. Paterson, E. Fodor, Emerging roles for the influenza A virus nuclear export protein (NEP). PLOS Pathog. 8, e1003019 (2012). 23236273 10.1371/journal.ppat.1003019 PMC3516560

[17] R. M. Krug, Functions of the influenza A virus NS1 protein in antiviral defense. Curr. Opin. Virol. 12, 1–6 (2015). 25638592 10.1016/j.coviro.2015.01.007 PMC4470714

[18] L. Zhang, Y. Shao, Y. Wang, Q. Yang, J. Guo, G. F. Gao, T. Deng, Twenty natural amino acid substitution screening at the last residue 121 of influenza A virus NS2 protein reveals the critical role of NS2 in promoting virus genome replication by coordinating with viral polymerase. J. Virol. 98, e0116623 (2024). 38054704 10.1128/jvi.01166-23 PMC10804943

[19] L. Zhang, Y. Wang, Y. Shao, J. Guo, G. F. Gao, T. Deng, Fine regulation of influenza virus RNA transcription and replication by stoichiometric changes in viral NS1 and NS2 proteins. J. Virol. 97, e0033723 (2023). 37166301 10.1128/jvi.00337-23 PMC10231140

[20] N. C. Robb, M. Smith, F. T. Vreede, E. Fodor, NS2/NEP protein regulates transcription and replication of the influenza virus RNA genome. J. Gen. Virol. 90, 1398–1407 (2009). 19264657 10.1099/vir.0.009639-0

[21] L. Brunotte, J. Flies, H. Bolte, P. Reuther, F. Vreede, M. Schwemmle, The nuclear export protein of H5N1 influenza A viruses recruits Matrix 1 (M1) protein to the viral ribonucleoprotein to mediate nuclear export. J. Biol. Chem. 289, 20067–20077 (2014). 24891509 10.1074/jbc.M114.569178 PMC4106323

[22] B. Mänz, L. Brunotte, P. Reuther, M. Schwemmle, Adaptive mutations in NEP compensate for defective H5N1 RNA replication in cultured human cells. Nat. Commun. 3, 802 (2012). 22549831 10.1038/ncomms1804

[23] S. Huang, J. Chen, Q. Chen, H. Wang, Y. Yao, J. Chen, Z. Chen, A second CRM1-dependent nuclear export signal in the influenza A virus NS2 protein contributes to the nuclear export of viral ribonucleoproteins. J. Virol. 87, 767–778 (2013). 23115280 10.1128/JVI.06519-11 PMC3554077

[24] R. E. O’Neill, J. Talon, P. Palese, The influenza virus NEP (NS2 protein) mediates the nuclear export of viral ribonucleoproteins. EMBO J. 17, 288–296 (1998). 9427762 10.1093/emboj/17.1.288 PMC1170379

[25] G. Neumann, M. T. Hughes, Y. Kawaoka, Influenza A virus NS2 protein mediates vRNP nuclear export through NES-independent interaction with hCRM1. EMBO J. 19, 6751–6758 (2000). 11118210 10.1093/emboj/19.24.6751 PMC305902

[26] M. Fukuda, S. Asano, T. Nakamura, M. Adachi, M. Yoshida, M. Yanagida, E. Nishida, CRM1 is responsible for intracellular transport mediated by the nuclear export signal. Nature 390, 308–311 (1997). 9384386 10.1038/36894

[27] K. Martin, A. Heleniust, Nuclear transport of influenza virus ribonucleoproteins: The viral matrix protein (M1) promotes export and inhibits import. Cell 67, 117–130 (1991). 1913813 10.1016/0092-8674(91)90576-k

[28] G. Whittaker, M. Bui, A. Helenius, Nuclear trafficking of influenza virus ribonuleoproteins in heterokaryons. J. Virol. 70, 2743–2756 (1996). 8627748 10.1128/jvi.70.5.2743-2756.1996 PMC190131

[29] F. Baudin, I. Petit, W. Weissenhorn, R. W. Ruigrok, In vitro dissection of the membrane and RNP binding activities of influenza virus M1 protein. Virology 281, 102–108 (2001). 11222100 10.1006/viro.2000.0804

[30] C. Elster, K. Larsen, J. Gagnon, R. W. Ruigrok, F. Baudin, Influenza virus M1 protein binds to RNA through its nuclear localization signal. J. Gen. Virol. 78, 1589–1596 (1997). 9225034 10.1099/0022-1317-78-7-1589

[31] J. Sun, L. Kuai, L. Zhang, Y. Xie, Y. Zhang, Y. Li, Q. Peng, Y. Shao, Q. Yang, W.-X. Tian, J. Zhu, J. Qi, Y. Shi, T. Deng, G. F. Gao, NS2 induces an influenza A RNA polymerase hexamer and acts as a transcription to replication switch. EMBO Rep. 25, 4708–4727 (2024). 39026012 10.1038/s44319-024-00208-4 PMC11549089

[32] H. Akarsu, W. P. Burmeister, C. Petosa, I. Petit, C. W. Müller, R. W. Ruigrok, F. Baudin, Crystal structure of the M1 protein-binding domain of the influenza A virus nuclear export protein (NEP/NS2). EMBO J. 22, 4646–4655 (2003). 12970177 10.1093/emboj/cdg449 PMC212717

[33] E. C. Hutchinson, E. M. Denham, B. Thomas, D. C. Trudgian, S. S. Hester, G. Ridlova, A. York, L. Turrell, E. Fodor, Mapping the phosphoproteome of influenza A and B viruses by mass spectrometry. PLOS Pathog. 8, e1002993 (2012). 23144613 10.1371/journal.ppat.1002993 PMC3493474

[34] P. Cassonnet, C. Rolloy, G. Neveu, P.-O. Vidalain, T. Chantier, J. Pellet, L. Jones, M. Muller, C. Demeret, G. Gaud, F. Vuillier, V. Lotteau, F. Tangy, M. Favre, Y. Jacob, Benchmarking a luciferase complementation assay for detecting protein complexes. Nat. Methods 8, 990–992 (2011). 22127214 10.1038/nmeth.1773

[35] J. R. Keown, Z. Zhu, L. Carrique, H. Fan, A. P. Walker, I. Serna Martin, E. Pardon, J. Steyaert, E. Fodor, J. M. Grimes, Mapping inhibitory sites on the RNA polymerase of the 1918 pandemic influenza virus using nanobodies. Nat. Commun. 13, 251 (2022). 35017564 10.1038/s41467-021-27950-w PMC8752864

[36] B. Zhang, M. Liu, J. Huang, Q. Zeng, Q. Zhu, S. Xu, H. Chen, H1N1 influenza a virus protein NS2 inhibits innate immune response by targeting IRF7. Viruses 14, 2411 (2022). 36366509 10.3390/v14112411 PMC9694023

[37] X. Liu, C. Yang, Y. Hu, E. Lei, X. Lin, L. Zhao, Z. Zou, A. Zhang, H. Zhou, H. Chen, P. Qian, M. Jin, HIST1H1C regulates interferon-β and inhibits influenza virus replication by interacting with IRF3. Front. Immunol. 8, 350 (2017). 28392790 10.3389/fimmu.2017.00350 PMC5364133

[38] M. Hatta, Y. Hatta, J. H. Kim, S. Watanabe, K. Shinya, T. Nguyen, P. S. Lien, Q. M. Le, Y. Kawaoka, Growth of H5N1 influenza A viruses in the upper respiratory tracts of mice. PLOS Pathog. 3, e133 (2007). 17922570 10.1371/journal.ppat.0030133 PMC2000968

[39] J. H. Kim, M. Hatta, S. Watanabe, G. Neumann, T. Watanabe, Y. Kawaoka, Role of host-specific amino acids in the pathogenicity of avian H5N1 influenza viruses in mice. J. Gen. Virol. 91, 1284–1289 (2010). 20016035 10.1099/vir.0.018143-0 PMC2878586

[40] B. Mänz, M. Schwemmle, L. Brunotte, Adaptation of avian influenza A virus polymerase in mammals to overcome the host species barrier. J. Virol. 87, 7200–7209 (2013). 23616660 10.1128/JVI.00980-13 PMC3700283

[41] R. Salomon, J. Franks, E. A. Govorkova, N. A. Ilyushina, H.-L. Yen, D. J. Hulse-Post, J. Humberd, M. Trichet, J. E. Rehg, R. J. Webby, R. G. Webster, E. Hoffmann, The polymerase complex genes contribute to the high virulence of the human H5N1 influenza virus isolate A/Vietnam/1203/04. J. Exp. Med. 203, 689–697 (2006). 16533883 10.1084/jem.20051938 PMC2118237

[42] E. K. Subbarao, W. London, B. R. Murphy, A single amino acid in the PB2 gene of influenza A virus is a determinant of host range. J. Virol. 67, 1761–1764 (1993). 8445709 10.1128/jvi.67.4.1761-1764.1993 PMC240216

[43] A. Mehle, V. G. Dugan, J. K. Taubenberger, J. A. Doudna, Reassortment and mutation of the avian influenza virus polymerase PA subunit overcome species barriers. J. Virol. 86, 1750–1757 (2012). 22090127 10.1128/JVI.06203-11 PMC3264373

[44] M. M. Lutz IV, M. M. Dunagan, Y. Kurebayashi, T. Takimoto, Key role of the influenza A virus PA gene segment in the emergence of pandemic viruses. Viruses 12, 365 (2020). 32224899 10.3390/v12040365 PMC7232137

[45] M. Bui, A. Helenius, The role of nuclear import and export in influenza virus infection. Trends Cell Biol. 6, 67–71 (1996). 15157497 10.1016/0962-8924(96)81017-8

[46] M. Bui, E. G. Wills, A. Helenius, G. R. Whittaker, Role of the influenza virus M1 protein in nuclear export of viral ribonucleoproteins. J. Virol. 74, 1781–1786 (2000). 10644350 10.1128/jvi.74.4.1781-1786.2000 PMC111655

[47] J. Peukes, X. Xiong, S. Erlendsson, K. Qu, W. Wan, L. J. Calder, O. Schraidt, S. Kummer, S. M. V. Freund, H. G. Kräusslich, J. A. G. Briggs, The native structure of the assembled matrix protein 1 of influenza A virus. Nature 587, 495–498 (2020). 32908308 10.1038/s41586-020-2696-8 PMC7116405

[48] L. Selzer, Z. Su, G. D. Pintilie, W. Chiu, K. Kirkegaard, Full-length three-dimensional structure of the influenza A virus M1 protein and its organization into a matrix layer. PLOS Biol. 18, e3000827 (2020). 32997652 10.1371/journal.pbio.3000827 PMC7549809

[49] K. Zhang, Z. Wang, X. Liu, C. Yin, Z. Basit, B. Xia, W. Liu, Dissection of influenza A virus M1 protein: pH-dependent oligomerization of N-terminal domain and dimerization of C-terminal domain. PLOS ONE 7, e37786 (2012). 22655068 10.1371/journal.pone.0037786 PMC3360003

[50] E. Hoffmann, G. Neumann, Y. Kawaoka, G. Hobom, R. G. Webster, A DNA transfection system for generation of influenza A virus from eight plasmids. Proc. Natl. Acad. Sci. U.S.A. 97, 6108–6113 (2000). 10801978 10.1073/pnas.100133697 PMC18566

[51] E. Fodor, M. Crow, L. J. Mingay, T. Deng, J. Sharps, P. Fechter, G. G. Brownlee, A single amino acid mutation in the PA subunit of the influenza virus RNA polymerase inhibits endonucleolytic cleavage of capped RNAs. J. Virol. 76, 8989–9001 (2002). 12186883 10.1128/JVI.76.18.8989-9001.2002 PMC136441

[52] E. Fodor, L. Devenish, O. G. Engelhardt, P. Palese, G. G. Brownlee, A. García-Sastre, Rescue of influenza A virus from recombinant DNA. J. Virol. 73, 9679–9682 (1999). 10516084 10.1128/jvi.73.11.9679-9682.1999 PMC113010

[53] I. Haralampiev, S. Prisner, M. Nitzan, M. Schade, F. Jolmes, M. Schreiber, M. Loidolt-Krüger, K. Jongen, J. Chamiolo, N. Nilson, F. Winter, N. Friedman, O. Seitz, T. Wolff, A. Herrmann, Selective flexible packaging pathways of the segmented genome of influenza A virus. Nat. Commun. 11, 4355 (2020). 32859915 10.1038/s41467-020-18108-1 PMC7455735

[54] A. V. Orjalo Jr., H. E. Johansson, Stellaris® RNA fluorescence in situ hybridization for the simultaneous detection of immature and mature long noncoding RNAs in adherent cells. Methods Mol. Biol. 1402, 119–134 (2016). 26721487 10.1007/978-1-4939-3378-5_10

[55] N. Tsanov, A. Samacoits, R. Chouaib, A.-M. Traboulsi, T. Gostan, C. Weber, C. Zimmer, K. Zibara, T. Walter, M. Peter, E. Bertrand, F. Mueller, smiFISH and FISH-quant – A flexible single RNA detection approach with super-resolution capability. Nucleic Acids Res. 44, e165 (2016). 27599845 10.1093/nar/gkw784 PMC5159540

[56] A. Punjani, J. L. Rubinstein, D. J. Fleet, M. A. Brubaker, cryoSPARC: Algorithms for rapid unsupervised cryo-EM structure determination. Nat. Methods 14, 290–296 (2017). 28165473 10.1038/nmeth.4169

[57] P. Emsley, B. Lohkamp, W. G. Scott, K. Cowtan, Features and development of Coot. Acta Crystallogr. D Biol. Crystallogr. 66, 486–501 (2010). 20383002 10.1107/S0907444910007493 PMC2852313

[58] I. W. Davis, A. Leaver-Fay, V. B. Chen, J. N. Block, G. J. Kapral, X. Wang, L. W. Murray, W. B. Arendall III, J. Snoeyink, J. S. Richardson, D. C. Richardson, MolProbity: All-atom contacts and structure validation for proteins and nucleic acids. Nucleic Acids Res. 35, W375–W383 (2007). 17452350 10.1093/nar/gkm216 PMC1933162

[59] A. J. Te Velthuis, N. C. Robb, A. N. Kapanidis, E. Fodor, The role of the priming loop in influenza A virus RNA synthesis. Nat. Microbiol. 1, 16029 (2016). 10.1038/nmicrobiol.2016.29 27572643

[60] G. Young, N. Hundt, D. Cole, A. Fineberg, J. Andrecka, A. Tyler, A. Olerinyova, A. Ansari, E. G. Marklund, M. P. Collier, S. A. Chandler, O. Tkachenko, J. Allen, M. Crispin, N. Billington, Y. Takagi, J. R. Sellers, C. Eichmann, P. Selenko, L. Frey, R. Riek, M. R. Galpin, W. B. Struwe, J. L. P. Benesch, P. Kukura, Quantitative mass imaging of single biological macromolecules. Science 360, 423–427 (2018). 29700264 10.1126/science.aar5839 PMC6103225

[61] R. Asor, D. Loewenthal, R. van Wee, J. L. Benesch, P. Kukura, Mass photometry. Annu. Rev. Biophys. 54, 379–399 (2025). 40327438 10.1146/annurev-biophys-061824-111652

[62] F. Madeira, N. Madhusoodanan, J. Lee, A. Eusebi, A. Niewielska, A. R. N. Tivey, R. Lopez, S. Butcher, The EMBL-EBI Job Dispatcher sequence analysis tools framework in 2024. Nucleic Acids Res. 52, W521–W525 (2024). 38597606 10.1093/nar/gkae241 PMC11223882

[63] X. Robert, P. Gouet, Deciphering key features in protein structures with the new ENDscript server. Nucleic Acids Res. 42, W320–W324 (2014). 24753421 10.1093/nar/gku316 PMC4086106

