# Supplementary material for: Regulatory hotspot on the influenza A virus polymerase revealed through the structure of the NEP-polymerase complex
Source: Sci Adv. 2026 Jan 23;12(4):eaeb4073. doi: 10.1126/sciadv.aeb4073 (PMC12829568; doi:10.1126/sciadv.aeb4073)
Supplement: Supplementary file 1 — Figs. S1 to S9 Tables S1 and S2 Legend for movie S1 Legend for dataset S1 [file sciadv.aeb4073_sm.pdf]

Science Advances
AAAS

Supplementary Materials for

Regulatory hotspot on the influenza A virus polymerase revealed through the structure of the NEP-polymerase complex

Alison Rep *et al.*

Corresponding author: Loïc Carrique, loic.carrique@strubi.ox.ac.uk;
Jonathan M. Grimes, jonathan.grimes@strubi.ox.ac.uk; Ervin Fodor, ervin.fodor@path.ox.ac.uk

Sci. Adv. **12**, eaeb4073 (2026)
DOI: 10.1126/sciadv.aeb4073

The PDF file includes:

Figs. S1 to S9
Tables S1 and S2
Legend for movie S1
Legend for dataset S1

Other Supplementary Material for this manuscript includes the following:

Movie S1
Dataset S1

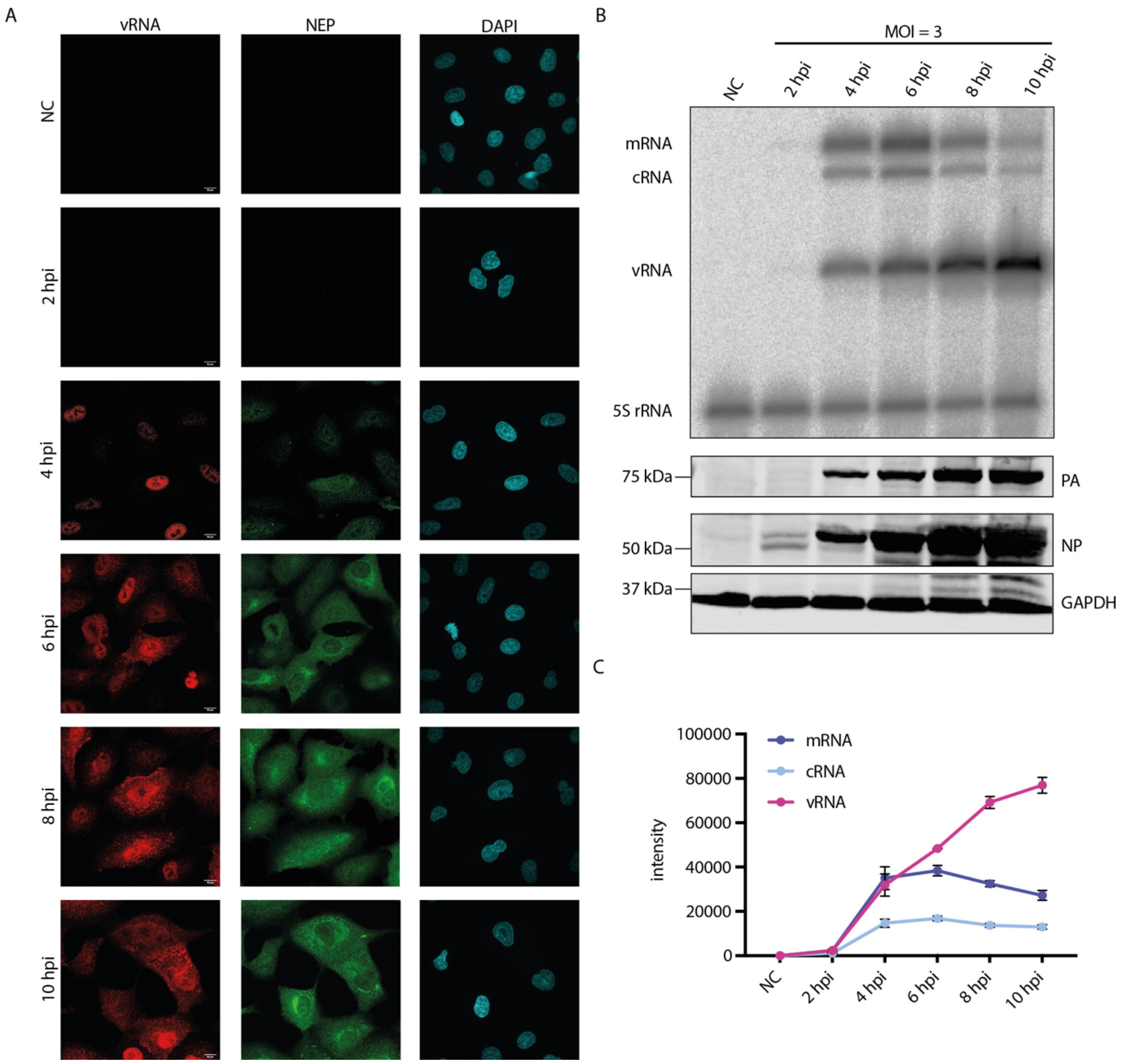

Fig. S1. The onset of nuclear export of vRNPs coincides with the accumulation of NEP. A549 cells were infected with A/WSN/33 (H1N1) influenza A virus at a multiplicity of infection of three, and samples were collected at two-, four-, six-, eight-, and ten-hours post-infection. **(A)** vRNP localisation was assessed using smFISH probes targeting NA vRNA, and NEP levels were determined by immunofluorescence using an antibody against NEP. DAPI staining was used to indicate nuclei. **(B)** The accumulation of viral RNAs and proteins was examined by primer extension assay and western blot analysis. **(C)** The levels of viral RNAs at different time points were quantified by measuring the band intensities from (B), normalised to 5S rRNA, and corrected by subtracting the background signal from the negative control (NC). Error bars represent the range of the mean of two independent biological replicates (n = 2). Scale bar: 10 μm.

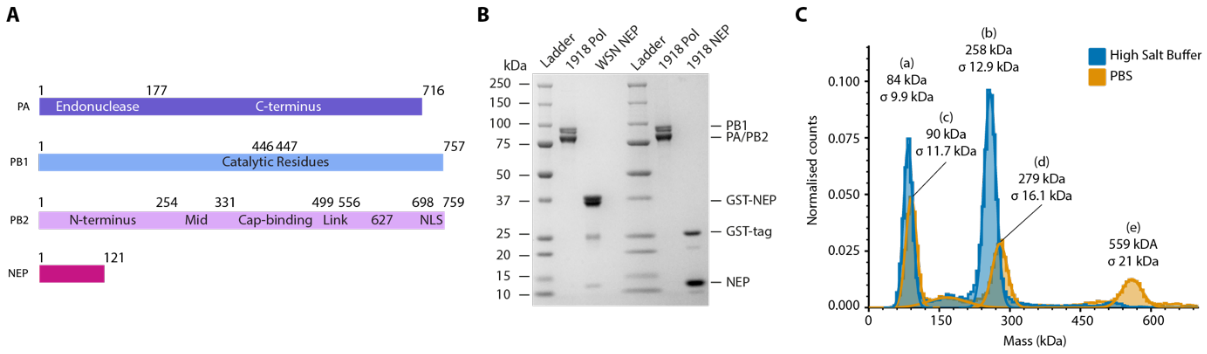

Fig. S2. Formation of the NEP-polymerase complex. (A) Schematic depicting domain structure of the polymerase heterotrimer subunits PA, PB1 and PB2, and NEP. **(B)** SDS-PAGE analysis of purified polymerase and NEP samples stained with Coomassie Brilliant Blue. **(C)** Mass photometry analysis of 1918 polymerase and WSN GST-NEP mixed in high salt buffer (blue), or in low salt buffer (orange) at a 1:1 molar ratio and 25 nM concentration. A dimeric GST-NEP complex can be observed in both the high salt buffer (a) and in PBS (c). A mass shift to a larger molecular weight can be observed in the monomeric polymerase population in PBS (d) compared to in the high salt buffer (b). In PBS, an additional population is observed with a molecular weight corresponding to a polymerase dimer with one or two copies of GST-NEP (e).

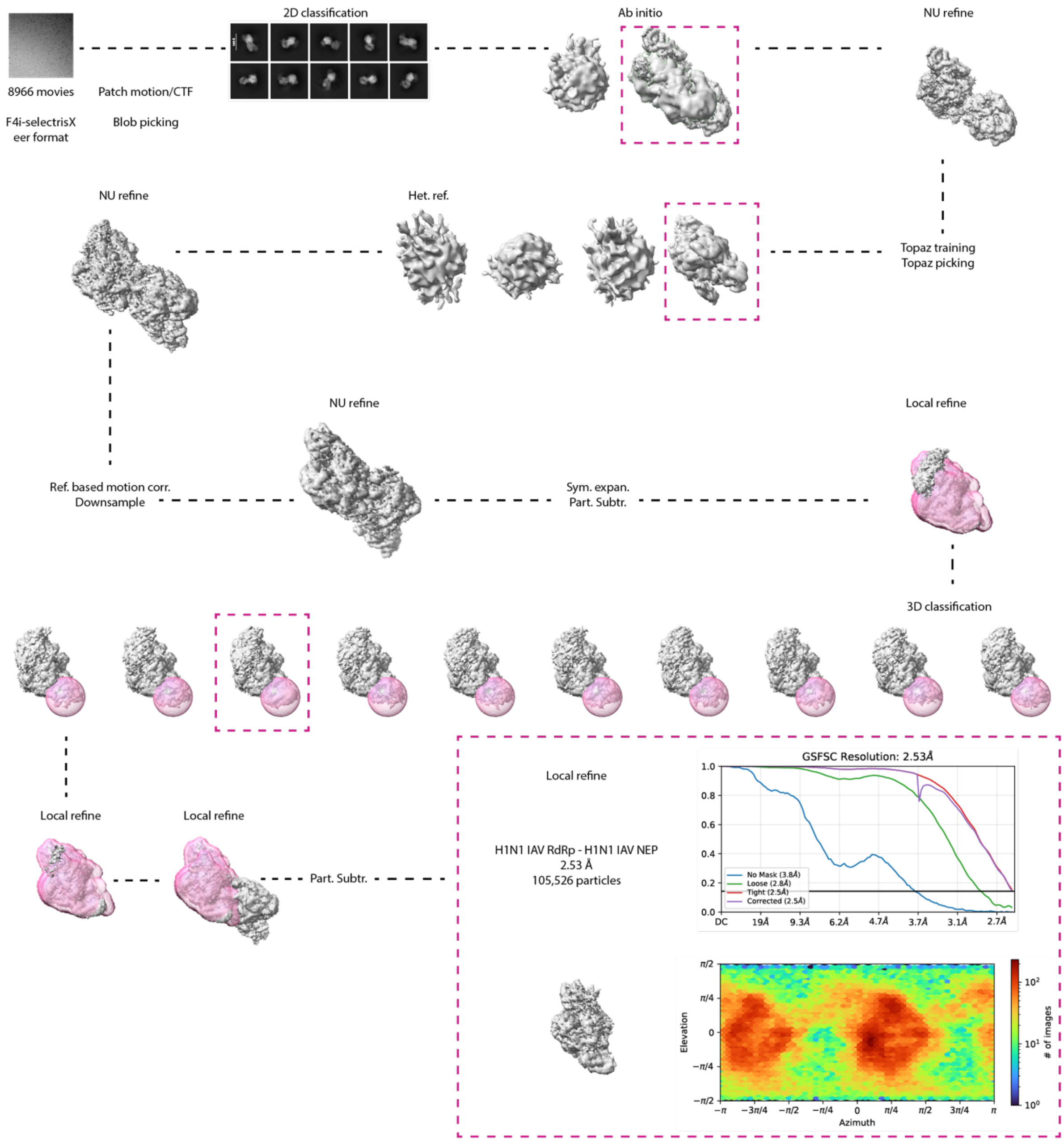

Fig. S3. Data collection, processing and analysis scheme of the A/Brevig Mission/1/18 (H1N1) polymerase and NEP complex. Pink globular shapes correspond to volume masks used for focused refinement.

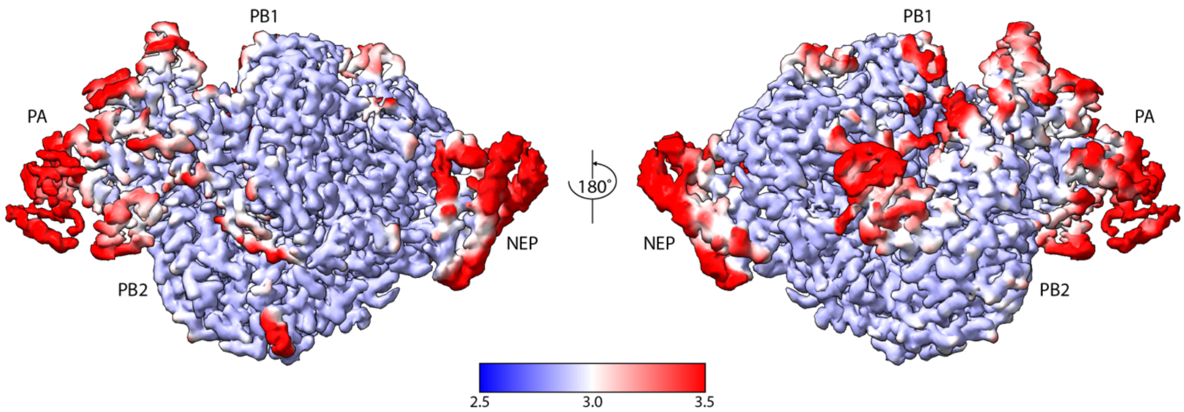

Fig. S4. Cryo-EM density map of NEP-polymerase complex. Volume surface is colour-coded by local resolution (see key), with shading corresponding to 3.0 Å resolution (blue) through 3.5 Å resolution (red). The NEP helices are clearly visible at this map threshold.

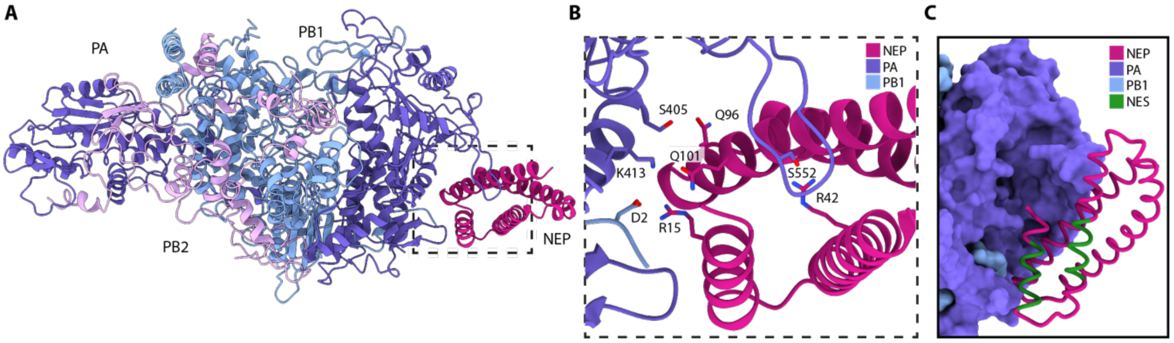

Fig. S5. Structure of the A/WSN/33 (H1N1) NEP bound to the pandemic A/Brevig Mission/1/18 (H1N1) influenza virus polymerase. (A) Cartoon model of the NEP-polymerase complex. Dashed rectangles denote the close-up view positions seen in (B). **(B)** Close-up views of NEP-polymerase complex. **(C)** Cartoon depiction of NEP bound to the polymerase shown in surface representation with the NES sequences of NEP shown in green.

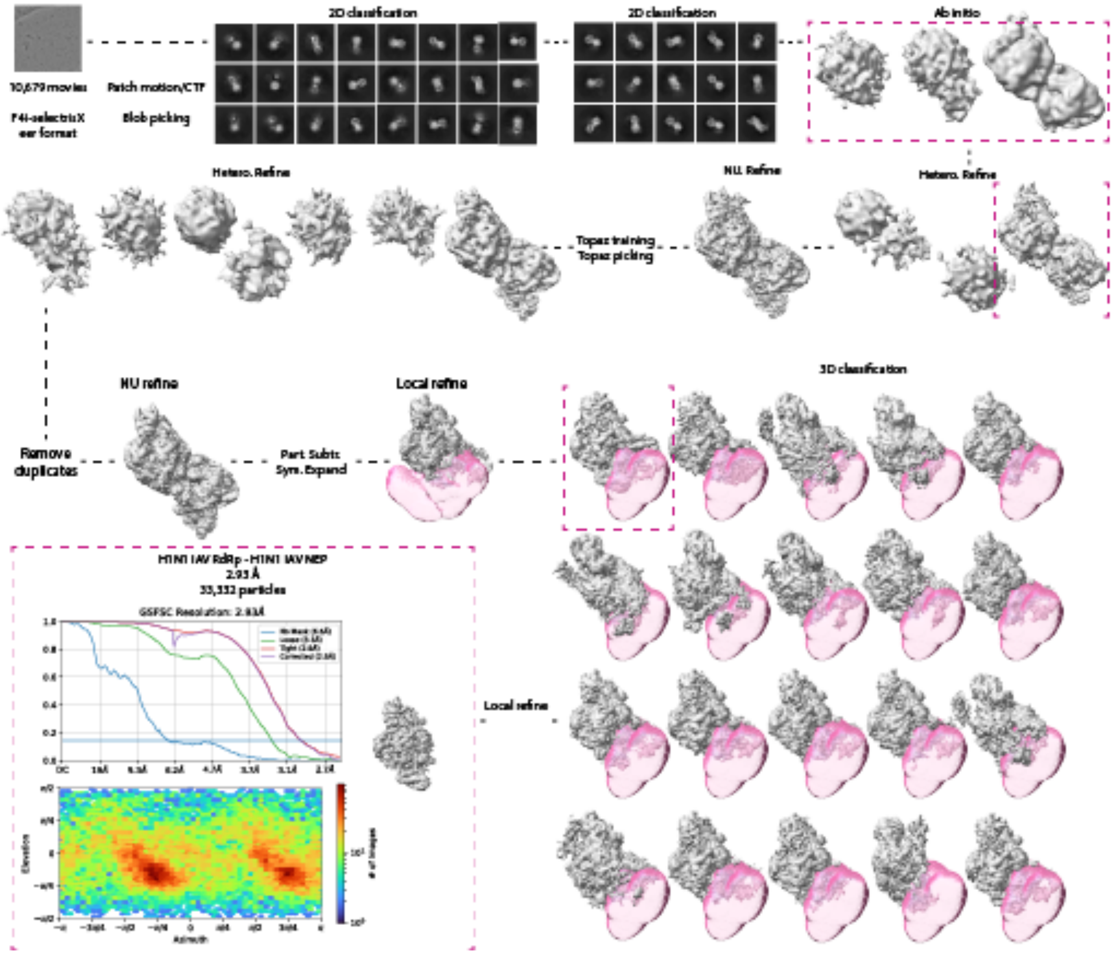

Fig. S6. Data collection, processing and analysis scheme of the A/Brevig Mission/1/18 (H1N1) polymerase and A/WSN/33 (H1N1) NEP complex. Pink globular shapes correspond to volume masks used for focused refinement.

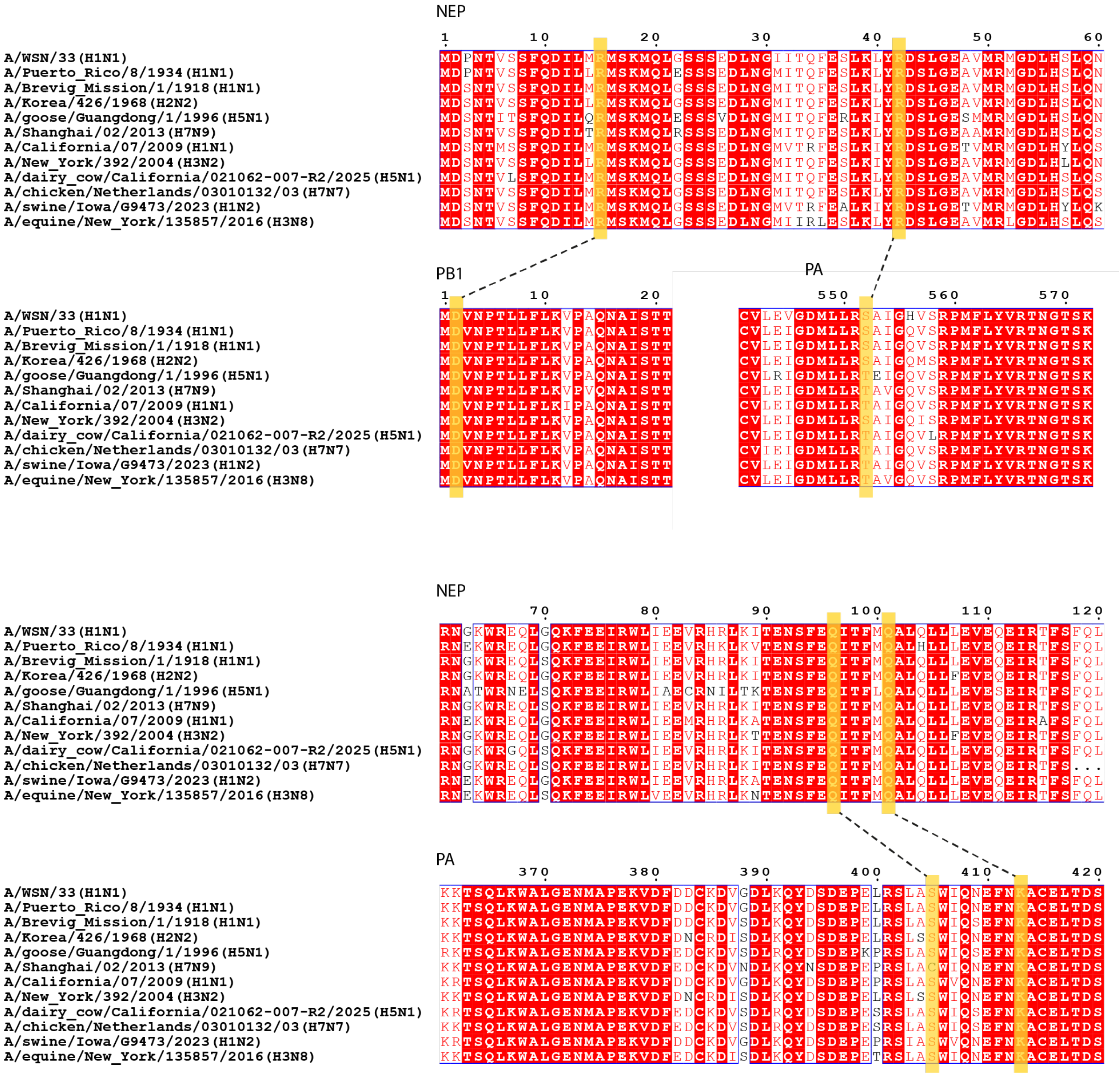

Fig. S7. The NEP-polymerase interaction interface is highly conserved across different influenza A virus subtypes. NEP, PB1 and PA amino acid sequences were downloaded from GISAID database and subjected to multiple sequence alignment using Clustal Omega. The results were visualised using ESPript 3.0. Alignment results of NEP and the corresponding PB1/PA residues are highlighted in yellow boxes.

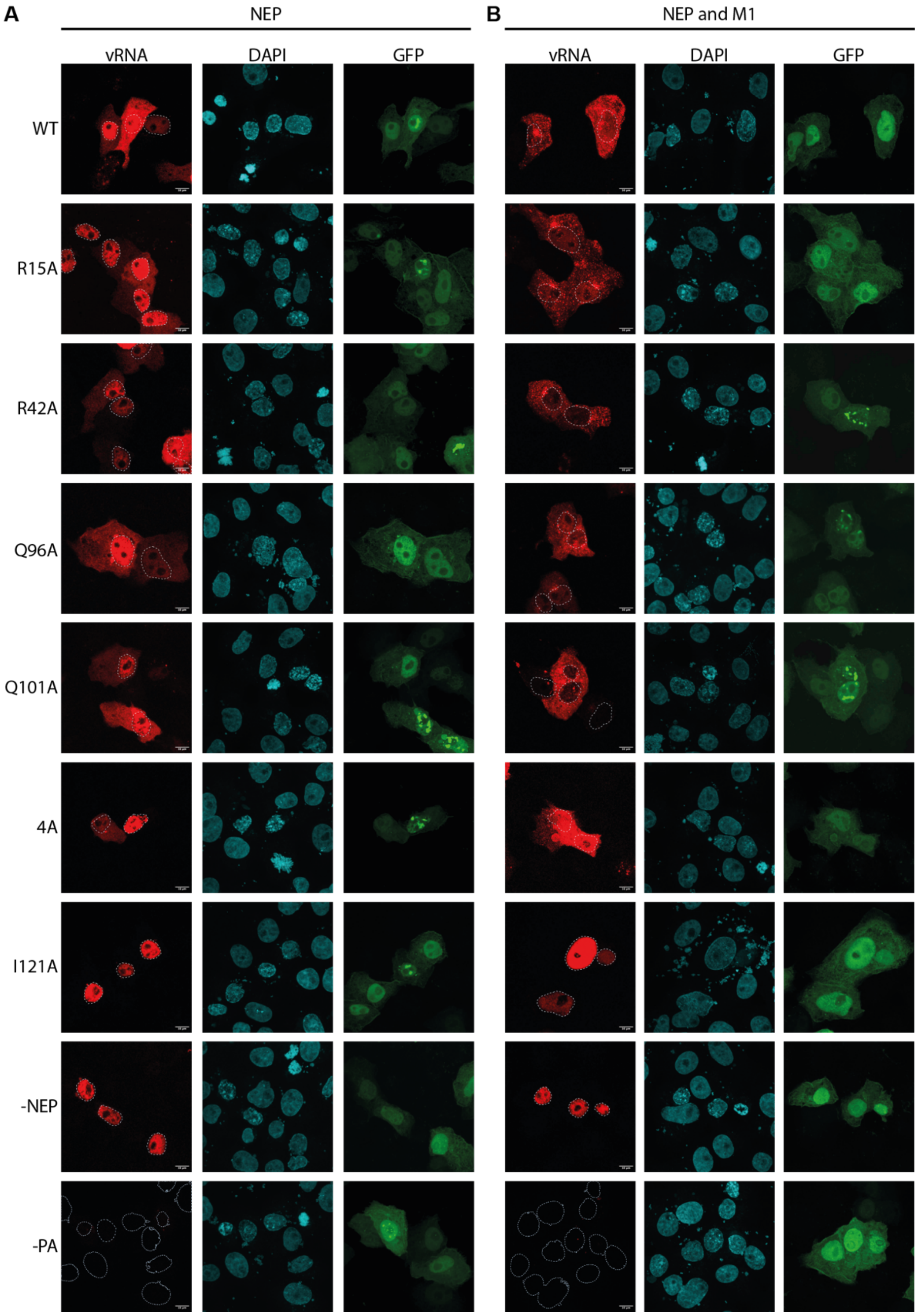

Fig. S8. Effect of NEP mutations on vRNP and NEP localisation. (A) Extended data corresponding to Fig. 4 with additional micrographs corresponding to DAPI staining and GFP signal. Vero E6 cells were transfected with vRNP reconstitution components in the presence of GFP-tagged wild-type (WT) NEP, an interface mutant NEPs, or in the absence of NEP (−NEP). In the −NEP condition, pcDNA-GFP was used as a replacement. A negative control lacking pCAGGS-PA (−PA) was also included. vRNP localisation was assessed using smFISH probes targeting NA vRNA. Cellular and nuclear boundaries were visualised using GFP fluorescence and DAPI staining, respectively. Scale bar: 10 µm. **(B)** Same as (A), with the addition of M1.

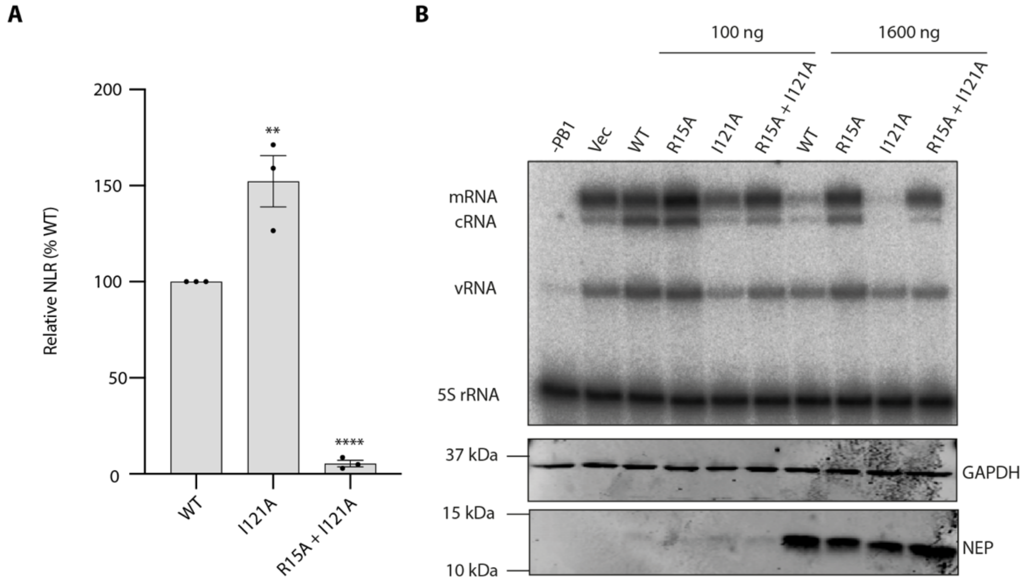

Fig. S9. Effect of the NEP I121A mutation on NEP-polymerase interactions and viral RNA synthesis. (A) The effect of the NEP I121A mutation alone or in combination with the NEP R15A mutation on NEP-polymerase interaction was analysed in a split-luciferase complementation assay. NLR values were normalised to the value of wildtype NEP (WT), set as 100%. Graphs represent the NLR or relative NLR of three independent biological replicates (mean ± s.e.m, n = 3). Significance was determined using ordinary one-way ANOVA with multiple comparisons relative to WT, ** $P \leq 0.01$, **** $P \leq 0.0001$. **(B)** Primer extension analysis of viral RNAs from vRNP reconstitution assays in the presence of low (100 ng) or high concentrations (1600 ng) of wildtype, mutant NEP (I121A or R15A + I121A), or vector only (Vec). Omission of PB1 (-PB1) served as a negative control and 5S rRNA was used as a loading control. Western blotting using antibodies against NEP and GAPDH was conducted to assess protein levels of NEP and GAPDH (loading control).

Table S1. Probes against the NA vRNA of A/WSN/33 (H1N1).

	DNA Probes (5' to 3')
**1**	GTTCACCATTGACAAGTAGT
**2**	TACTGTAGATTGGTCTTGGC
**3**	GAGGACGCAATCTGGACTAG
**4**	ACAGGGCTAGACTGTATGAG
**5**	AGTTTCGTTCAACATCCTGA
**6**	TTGTGGCAATGACTGATCGG
**7**	GGATCCTAATGGATGGACAG
**8**	CCAGACATGGGTTTGAGATG
**9**	GGTGTTTGGATAGGAAGGAC
**10**	TGCTGATGGAGCAAACGGAG
**11**	CCAAAGATGGAACAGGCAGC
**12**	TAGGATACATCTGCAGTGGG
**13**	CCTTCGACCAAAACCTAGAT
**14**	TGCACCTAATTCTCACTACG
**15**	TCGAGAAGGGGAAGGTTACT
**16**	GCTGGCCTCGTACAAAATTT
**17**	TTACCATAATGACCGATGGC
**18**	GAGTCTGAATGTACCTGTGT
**19**	AATGGGCTGGCTAACAATCG
**20**	GTCAGCAAGTGCATGTCATG
**21**	CCCGTACAATTCAAGGTTTG
**22**	TTATAGGGCCTTAATGAGCT
**23**	GGGGACCTTTAAGGACAGAA
**24**	CGCCTTACTGAATGACAAGC
**25**	GAATGCAGGACCTTTTTTCT
**26**	TGGTTCCAAAGGAGACGTTT
**27**	GTGGGCTATACACAGCAAAG
**28**	TAACCGGCAATTCATCTCTT
**29**	CCTATAAAGTTGTTGCTGGG
**30**	GAATATGCAACCAAGGCAGC
**31**	AGCCATTCAATTCAAACCGG
**32**	GGATCGATCTGTATGGTAGT
**FLAP**	ATTO550/CTGAGTCCAGCTCGAAACTTAGGAGG/Cy3

Table S2. Cryo-EM analysis data parameters. Parameters are included for data collection, EM data processing and model building and validation.

	EMD-54287	EMD-54376
	9RVE	9RYC
**Data collection**		
Microscope	Titan Krios G3i (OPIC)	Titan Krios G3i (OPIC)
Voltage (kV)	300	300
Detector	Falcon 4i - SelectrisX	Falcon 4i - SelectrisX
Recording mode	eer	eer
Magnification	130,000	130,000
Movie/micrograph pixel size (Å)	0.932	0.932
Dose rate (e-/px/sec)	9.15	9.27
Number of frames per movie	60	60
Movie exposure time (s)	6.29	6.2
Total dose (e-/Å^2)	50	50
Defocus range (um)	1.4 to 2.4	1.4 to 2.4
**EM data processing**		
Number of movies/micrographs	8,966	10,675
Box size (px)	400	400
Particle number (After initial 3D Classes)	430K	586K
Particle number (used in final map)	105k	28K
Symmetry	C1	C1
Map resolution (Å, FSC 0.143)	2.53	2.93
Local resolution range (Å, FSC 0.5)	2.48 - 30	2.74 - 30
Map sharpening B-factor (Å^2)	61	40
**Model Building and Validation**		
Initial model used	8R1J	9RVE
Model composition		
Non-hydrogen protein atoms	29189	28022
Protein residues	1811	1797
Nucleotides	/	/
B factors (Å^2) - min/max/mean		
Protein	22.60/143.28/62.69	38.38/155.21/73.12
Nucleotide	/	/
Ligand	/	/
RMSD from ideal		
Bond length (Å)	0.004	0.003
Bond angles (°)	0.578	0.566
Validation		
Molprobity score	1.66	1.84
Clashscore	5.79	8.97
Rotamers outliers (%)	0	0
FSC (0.5) model-vs-map	2.7	3.2
CC model-vs-map (masked)	0.78	0.79
Ramachandran plot		
Favored (%)	94.87	94.82
Allowed (%)	4.92	5.07
Outliers (%)	0.11	0.11

Captions for supplementary movies

Movie S1. Structural comparison of polymerase bound to NEP, huANP32B and CTD of RNA polymerase II. The movie displays the structures of the polymerase bound to NEP (PDB 9RVE, this study), huANP32B (PDB 8R1J) or the CTD of RNA polymerase II (PDB 8R60) and zoom on the PA-C and PB1-N highlighting the PA-C/PB1-N interface as a hotspot for the binding of these major regulatory factors.

Captions for supplementary dataset

Dataset S1. Data values from the split-luciferase complementation assays.
